# Electrochemically reduced graphene oxide integrated with carboxylated-8-carboxamidoquinoline: A platform for highly sensitive voltammetric detection of Zn(II) ion by screen-printed carbon electrode

**DOI:** 10.1371/journal.pone.0315974

**Published:** 2025-02-07

**Authors:** Ling Ling Tan, Nur Syamimi Mohamad, Nurul Izzaty Hassan, Choo Ta Goh

**Affiliations:** 1 Southeast Asia Disaster Prevention Research Initiative (SEADPRI), Institute for Environment and Development (LESTARI), Universiti Kebangsaan Malaysia, Selangor Darul Ehsan, Malaysia; 2 Department of Chemical Sciences, Faculty of Science and Technology, Universiti Kebangsaan Malaysia, Selangor Darul Ehsan, Malaysia; Center for Research and Technology Transfer, VIET NAM

## Abstract

Zinc has been demonstrated to boost immune response during SAR-CoV-2 infection, where it prevents coronavirus multiplication. Clinical investigations have testified to its beneficial effects on respiratory health and its deficiency may reduce immune function. A highly sensitive detection of Zn(II) ion via differential pulse voltammetry (DPV) utilizing an environmentally friendly modified screen-printed carbon electrode (SPCE) of electrochemically reduced graphene oxide (ErGO) embedded with carboxylated-8-carboxamidoquinoline (CACQ) as Zn(II) chelating ligand. The green CACQ/ErGO-modified SPCE was characterized by spectroscopy techniques, such as Fourier-transform infrared (FTIR) spectroscopy, Raman spectroscopy, and field-emission scanning electron microscopy with energy dispersive X-ray (FESEM-EDX). The modified electrode-solution interface was studied by electrochemical cyclic voltammetry (CV) and DPV methods. The CACQ-modified wrinkled ErGO electrode conferred a large surface-to-volume ratio with multiple binding sites resulting in greater opportunity for multiple dative covalent binding events with Zn(II) via coordination chemistry, and considerably accelerated the electron transfer rate at the electrode surface. The green Zn(II) sensor demonstrated a quick response time (60 s), broad linear range [1 pM-1 μM Zn(II) ion], a limit of detection (LOD) of 0.53 pM, 35 days of storage period (≥80% of its initial response retained), good reproducibility [relative standard deviation (RSD) = 3.4%], and repeatability (RSD = 4.4%). The developed electrode was applied to determine Zn(II) ion concentration in dietary supplement samples, and the results were in good agreement with those obtained from inductively coupled plasma-mass spectrometry (ICP-MS).

## Introduction

Zinc (Zn) is an essential mineral that is found in every cell in the human body [1]. It is involved in over 300 different enzymes that are responsible for a variety of functions, including cell growth and division, protein synthesis, and DNA replication [2, 3]. Zn is also important for the immune system [4, 5]. It helps to produce white blood cells and antibodies, which are essential for fighting infections [6]. Besides, other research has discovered that consuming up to 150 mg of Zn every day is necessary to reduce viral infections [7]. In addition, Zn is involved in the production of sex hormones, including testosterone and estrogen [8]. The acceptable upper intake level (UL) for Zn has been established at 40 mg per day by the Institute of Medicine (IOM), USA. However, the Recommended Dietary Allowance (RDA) for adults is between 8 mg and 11 mg [9]. Yet, Zn deficiency can lead to several health problems, including anemia [10], poor wound healing ability [11], and impaired cognitive function [12]. So, it is important to be able to detect Zn(II) ions in biological samples.

Various instrumental methods have been employed for the detection of Zn(II) ion, including atomic absorption spectroscopy (AAS) [13], inductively coupled plasma-mass spectrometry (ICP-MS) [14], and inductively coupled plasma-atomic emission spectrometry (ICP-AES) [15]. In recent years, the use of ultraviolet photoelectron spectroscopy (UV-PS) has become a popular technique for the detection of Zn-related materials [16]. UV-PS is based on the principle that electrons are ejected from atoms when they absorb light in the ultraviolet (UV) portion of the electromagnetic spectrum. The strength of this emission can be used to determine the concentration of the Zn(II) ion [17]. However, all of these techniques require expensive and high-resolution instrumentation.

Graphene, a planar sheet of single-atom-thick *sp*^2^-hybridized carbon nanostructure networking arranged in a honeycomb lattice [18, 19] has sparked tremendous attention due to its exceptional properties, including its high surface-to-volume ratio, optically transparency, superior thermal conductivity, and excellent mechanical stability [20]. In addition to being flexible and tougher than diamond, these two-dimensional (2-D) nanostructures of carbon also conduct electricity more quickly than any other material at room temperature. As a result, graphene is now used in a wide range of applications e.g., technological devices [21], pharmaceutical delivery systems [22], capacitors [23], and electrochemical sensors [24]. However, difficulties in formulating plans for massive graphene production have stymied scientific advancement in this area [20].

The chemical reduction of graphene oxide (GO) is one of the numerous methods for manufacturing graphene and is thought to be the most cost-effective method for producing graphene-based in large quantities [25]. By removing various oxygen-containing functional groups (i.e., ketone carbonyls, epoxides, alcohols, and carboxylic groups) created during the chemical treatment of the graphite [26] and reinstating the conjugation of the C = C double bond (aromaticity) [27], the reduced graphene oxide (rGO) regains graphene-like properties, such as a strong π-electronic configuration, becomes less hydrophilic, and has a better conductivity [28, 29]. Additionally, rGO-graphenoids can be made using chemical, thermal, or electrochemical methods [30, 31]. Thus, rGO is now developing into a very good pristine graphene/GO compromise. Nonetheless, chemical reduction processes may utilize too many reducing agents, which could contaminate the finished product and even be hazardous to human health and the environment [32, 33]. The thermal reduction technique, on the other hand, uses high temperatures to eliminate the covalently-bonded oxygen, which results in long, tedious, and expensive work-ups [34].

Further, immobilization of the Zn(II) probe is an essential aspect of sensor development. 6-methoxy-(8-p-toluenesulfonam-ido)quinoline (TSQ) has proven to be the most effective probe for zinc recognition in an aqueous medium [35]. The TSQ derivatives-based sensor, however, sustained lower analytical performance for quantifying free Zn(II) ions in cells because the TSQ derivatives have weak water solubility and membrane permeability [36]. Several initiatives have been made to increase the water solubility properties of TSQ, including switching 8-aminoquinoline (8-AQ) to 8-carboxamidoquinoline (8-CQ)-based parent structure [37–43]. Additionally, based on our prior analysis, it was feasible to deduce those 8-CQ derivatives with tremendous promise as useful Zn(II) ion receptors due to their great selectivity, strong reactivity, and biocompatibility properties [44].

**Fig 1:**
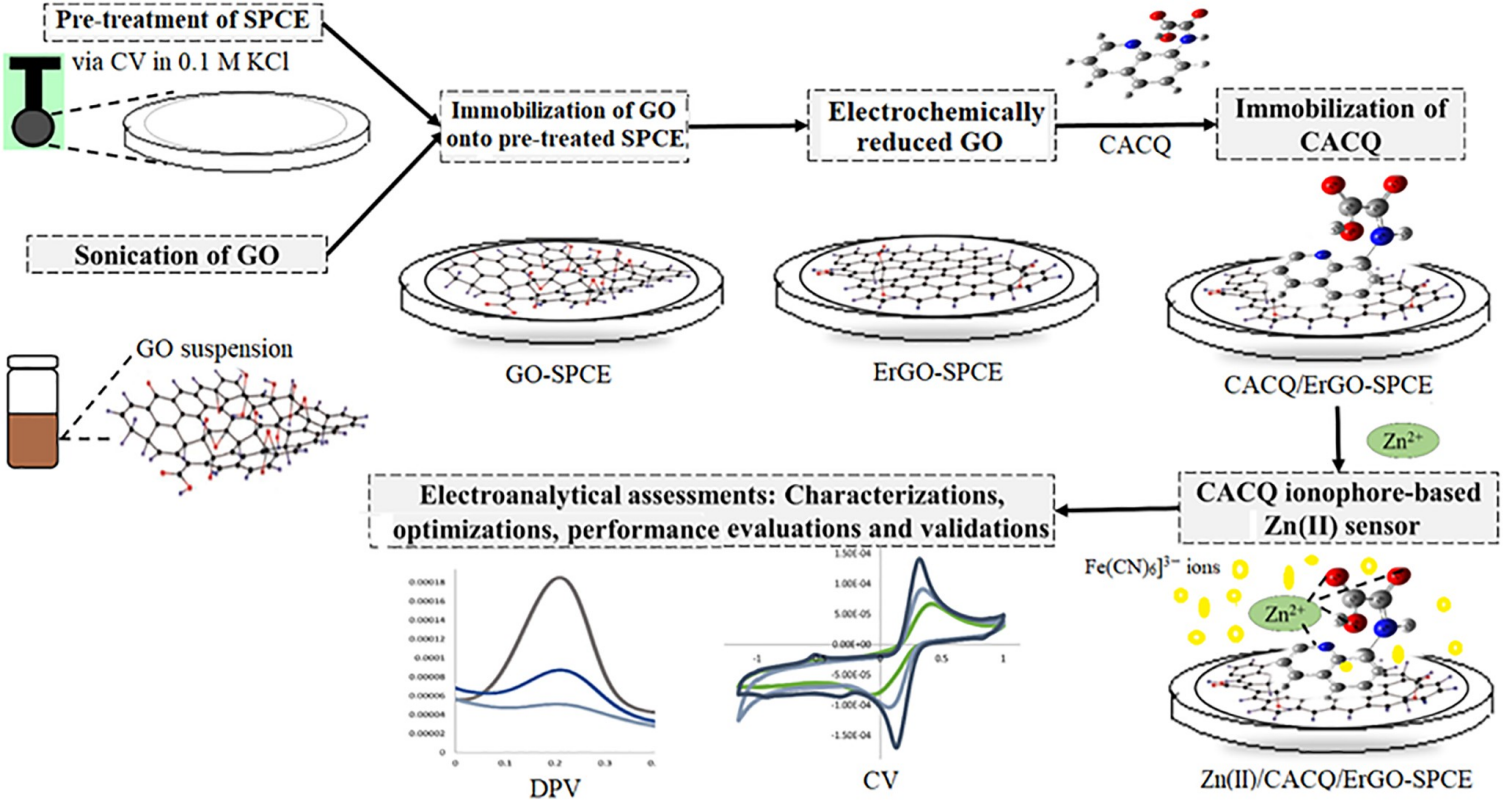
The schematic design of Zn(II) ion sensor based on an electrochemical modification of screen-printed carbon electrode surface with graphene sheets integrated with quinoline derivative chelating ligand in the presence of K_3_[Fe(CN)_6_] redox indicator.

In this study, an electrochemically reduced graphene oxide (ErGO) approach, which is environmentally friendly, quick, simple, harmless, and time-efficient was applied to modify the SPCE surface. The graphene-based sheets were initially deposited and adsorbed on the pre-treated SPCE surfaces through both electrostatic and π−π interactions between the electrode and GO. The electrodeposition of GO via cyclic voltammetry (CV) approach was then conducted for several cycles to produce an ErGO-modified SPCE electrode. The carboxylated-8-CQ (CACQ) as the ionophore for Zn(II) ion detection was subsequently applied to these surfaces via π−π stacking interactions between the aromatic ring tethered and the hexagonal rings of the ErGO-graphenoids material. Thus, the use of ErGO offers an excellent conducting substrate for effective electron transfer as well as an immobilization platform for tying CACQ chelating agent to the SPCE. The schematic layout of this CACQ ionophore-based Zn(II) electrochemical sensor in the presence of K_3_[Fe(CN)_6_] redox mediator is illustrated in Fig 1.

## Materials and methods

### Materials and reagents

All the materials and reagents used in this investigation were of analytical grade and were utilized directly as received, with no further purification. Zinc sulphate heptahydrate (ZnSO_4_·7H_2_O) from Sigma-Aldrich (St. Louis, Missouri, United States) was used to prepare a stock solution of Zn(II) ions. Tris(hydroxymethyl)aminomethane (Tris, ACS reagent, ≥99.8%), sulphuric acid (H_2_SO_4_, 95–98%), nitric acid (HNO_3_, ACS reagent, 65% w/w), potassium permanganate (KMnO_4_, ≥99%), hydrogen peroxide solution (H_2_O_2_, 30% w/w), and hydrochloric acid (HCl, 37%) were all sourced from Sigma-Aldrich from St. Louis, Missouri, United States. Other chemicals and reagents were obtained from R&M Chemicals, Semenyih, Selangor, Malaysia. De-ionized water (DIW) with a resistivity of at least 18.2 MΩ·cm was used to prepare all solutions. Additionally, a 0.1 M potassium chloride (KCl) solution was used for the pre-treatment of S-U07-5004610 screen-printed carbon electrodes (SPCE) and electrochemically reduced graphene oxide (ErGO). A 10 mM potassium ferricyanide (K_3_[Fe(CN)_6_]) solution, prepared in 0.1 M KCl, was utilized as an electron transfer agent for electrochemical measurements. K_3_[Fe(CN)_6_] was provided by Nacalai Tesque (Nakagyo-ku, Kyoto), KCl was purchased from BDH Chemicals Ltd.

### Instrumentations

Attenuated Total Reflectance (ATR) spectra were acquired from the Agilent Cary 630 FTIR spectrometer in the wavenumber range of 4000–650 cm^-1^, 74 scans at 4 cm^–1^ resolution with a total measurement time of 30 seconds. ^1^H NMR spectrum of CACQ were recorded on a NMR spectrometer (Advance 400 III HD Bruker) at a magnetic field strength of 400.17 MHz. Measurements were performed at 22.85°C using deuterated DMSO as the solvent, with TMS, Si(CH_3_)_4_ as the internal reference. The sample concentration was 1 mM, and solvent signal suppression was applied where necessary. Detailed chemical shift (δ), the multiplicity, the integration, the coupling constants (in Hz), the type of proton and assignation of protons are provided in S1 Appendix. ^13^C NMR spectrum of CACQ were recorded on an NMR spectrometer (Advance 400 III HD Bruker) at a magnetic field strength of 100.63 MHz. Measurements were performed at 22.85°C using deuterated DMSO as the solvent, with TMS, Si(CH_3_)_4_ as the internal reference. The sample concentration was 1 mM, and solvent signal suppression was applied where necessary. Detailed chemical shifts (δ), and assignation of carbons are provided in S1 Appendix. The molecular mass spectrum was collected with a positive mode of Bruker MicroTof-Q (Bruker Daltonics). The pH was determined using a pH meter (pH meter Metrohm 827).

The surface morphology and elemental composition of the modified electrodes were investigated with a Zeiss Merlin/Merlin Compact/Supra 55VP field-emission scanning electron microscope with energy dispersive X-ray (FESEM-EDX). The acceleration voltage was set to 3.0 kV. In order to prepare the samples for FESEM, pre-treatment of the Screen-Printed Carbon Electrode (SPCE) surface was done before the modification with Graphene Oxide (GO) and Electrochemically Reduced Graphene Oxide (ErGO). Initially, 10 μL of the GO suspension was dispensed onto the pre-treated SPCE surface and allowed to dry under ambient conditions, resulting in a GO-modified SPCE (GO-SPCE). This GO-modified sample was directly utilized for FESEM analysis. For the ErGO-SPCE sample, the GO-SPCE underwent electrochemical reduction. The reduction process involved applying voltage sweeps restricted to 20 cycles between -1.5 and +0.5 V versus an Ag/AgCl reference electrode in a 0.1 M KCl solution at a scan rate of 100 mV/s. This procedure produced the reduced graphene oxide-modified SPCE (ErGO-SPCE). Subsequently, the ErGO-modified SPCE was allowed to dry at 25°C. The interface of the ErGO-modified electrode was then used for further characterization and analysis using FESEM.

Raman spectroscopy was performed using a Thermo Scientific DXR2xi Raman Microscope. The wavenumber range spanned from 50 cm^−1^ to 3500 cm^−1^, with a resolution of 2 cm^−1^. The laser excitation wavelength used for the analysis was 532 nm. Cyclic voltammetry (CV) and differential pulse voltammetry (DPV) were performed by Autolab PGSTST and NOVA 1.10 as data processing software. An open circuit of a three-electrode system that consists of a working electrode (WE, SPCE), counter electrode (CE, platinum), and reference electrode (RE, Ag/AgCl). CV was performed using potassium ferricyanide (K_3_[Fe(CN)_6_]) as the electrolyte. The potential was swept between -1.5 V and +0.5 V at a scan rate of 100 mV s^−1^. The SPCE working electrode was purchased from Scrint Technology (M) Sdn. Bhd.

An electronic melting point device (Manufacturer: Bibby, Model: Stuart^™^ melting point apparatus SMP10) was used to determine the melting point of the compound. After filling a capillary tube with around 5 mg of the sample, the tube was put into the device. The melting point was determined as the temperature at which the sample began to melt and turned entirely liquid. The temperature was raised at a rate of 1°C per minute. To assure accuracy, the experiment was conducted three times, and the average melting point was recorded. Real sample study of the CACQ ionophore-based Zn(II) sensor was verified using a Perkin Elmer NexION 2000 inductively coupled plasma-mass spectrometer (ICP-MS).

### Synthesis and characterization of zinc’s chelating ligand

Eq (1) simplifies the synthesis protocol of carboxylic acid-8-carboxamidoquinoline (CACQ), the chelating ligand of Zn(II) from ester-8-carboxamidoquinoline (ECQ) via a two-step process, i.e. acid-base reduction and liquid-liquid extraction. Briefly, the brown solid-ester compound of ECQ was hydrolyzed under reflux in a mixture of tetrahydrofuran/methanol (THF/MeOH) solution for one and a half hours [Eq (1a)], followed by a reduction reaction with 1 N base KOH, and acidified with 1 N acid HCl [Eq (1b)]. The resultant mixture was then extracted with ethyl acetate (CH_3_COOC_2_H_5_) using a partitioning technique [Eq (1c)], and the organic solvent was eliminated with a rotary evaporator in order to yield the CACQ, which appeared as a yellow solid-carboxylic acid compound [Eq (1d)]. The resulting CACQ product was subsequently examined by using a variety of spectroscopic methods, including ^1^H and ^13^C NMR, FTIR-ATR, and the positive ion mode of ESI-MS.


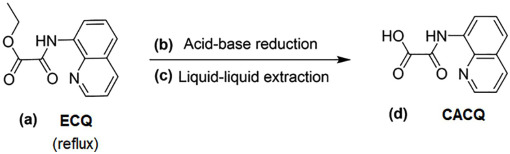

(1)


### Preparation of graphene oxide

2 g of graphite powder underwent pre-treatment by sonicating with 20 mL of 99.5% acetic acid for 5 h at room temperature (25°C). The solution was then filtered and rinsed with DIW until the filtrate reached a neutral pH, and the resulting graphite flakes were dried overnight at 40°C. Graphene oxide (GO) was synthesized via chemical oxidation of the pre-treated graphite flakes according to an adapted [45] method. A mixture of concentrated HNO_3_ and H_2_SO_4_ at a volume ratio of 1:3 was added slowly into the pre-treated graphite flakes in a 1 L conical flask and agitated gently for an hour in an ice bath. 6 g of KMnO_4_ was then gradually added and vigorous stirring persisted for three days in a silicone oil bath heated at 35°C. Next, 2.5% H_2_O_2_ solution was slowly added to cease the chemical oxidation reaction, and the mixture’s color changed to a vivid yellow, signifying a high amount of oxidation had taken place. The mixture was left overnight before decantation was performed by pipette-removing the supernatant. The resulting precipitates were then washed with 10% HCl two times, followed by thoroughly washing with DIW until pH 7 was achieved in the filtrate by using decantation and centrifugation techniques. The dark brown paste was then dried for an entire night in a vacuum oven to obtain a dark brown solid of GO.

### Fabrication of Zn(II) electrochemical sensor based on CACQ/ErGO-modified SPCE

Pre-treatment of the SPCE surface was conducted by scanning the electrode from 0.0 V to +1.5 V versus Ag/AgCl reference electrode in 0.1 M KCl at 100 mV s^-1^ scan rate for three cycles. Dark brown GO solution in DIW at 1 mg mL^-1^ was sonicated for an hour before dispensing some 10 μL of the GO suspension on the pre-treated SPCE surface, and allowed to dry at ambient conditions to produce GO-modified SPCE. A three-electrode system comprised of GO-SCPE (WE), platinum (CE), and Ag/AgCl (RE) was set up to accomplish the electrochemical reduction of GO. Electrochemically reduced graphene oxide (ErGO) via voltage sweeps was restricted to 20 cycles between -1.5 and +0.5 V versus Ag/AgCl reference electrode in 0.1 M KCl at a scan rate of 100 mV s^-1^. The ErGO-modified SPCE was then allowed to dry at 25°C. The ErGO-modified electrode-solution interface was studied by the electrochemical CV method. Next, an aliquot of 10 μL of the CACQ sensing reagent was applied on the surface of the ErGO electrode by dropping pipette technique and left to dry at ambient temperature. The CACQ-modified ErGO electrode was then immersed in a Zn(II) ion solution and allowed to dry prior to electroanalytical measurement with CV and DPV techniques in 0.1 M KCl supporting electrolyte containing 10 mM K_3_[Fe(CN)_6_].

The electrodynamic data of modified electrodes namely the anodic peak potential (*E*_pa_), cathodic peak potential (*E*_pc_), potential difference for oxidation and reduction of K_3_[Fe(CN)_6_] (Δ*E*_p_), anodic peak current (*i*_pa_), cathodic peak current (*i*_pc_), and anodic-to-cathodic peak current ratio (|*i*_pa_*/i*pc|), in 0.1 M KCl containing 10 mM K_3_[Fe(CN)_6_] are recorded and calculated. To enhance clarity and readability, we converted the original values into micro units (e.g., microamperes). This conversion naturally reduced the number of significant figures displayed, aligning with standard scientific reporting practices while maintaining the accuracy of the data.

### Optimization and electroanalytical assessment of Zn(II) sensor

The DPV profiles of the modified electrodes before and after the reaction with Zn(II) ion were acquired in the presence of 10 mM K_3_[Fe(CN)_6_] redox mediator in 0.1 M KCl within the potential range of 0.0 V to +0.5 V versus Ag/AgCl reference electrode. The ErGO-SPCEs modified with different concentrations of CACQ ligand compound between 0.2 mg mL^-1^ and 1.0 mg mL^-1^ were examined with the DPV electrochemical method for the determination of 0.1 M Zn(II) ion. pH effect on the Zn(II) electrochemical sensor response was carried out by reacting the CACQ/ErGO-SPCE with Zn(II) ion prepared in 0.1 M Tris buffer solution at different pHs (i.e. pH 5.0, pH 5.5, pH 6.0, pH 6.5, and pH 7.0), which were adjusted using 0.1 M HCl and 5 M NaOH solutions. A leaching test over the quinoline derivative chelating compound-immobilized ErGO electrode was performed by immersing the Zn(II) sensor into DIW for 10 min, and the DPV response of the sensor towards determination of 0.1 M Zn(II) at pH 6.5 was captured every 2 min at 0.22 V. Accumulation time of the Zn(II) sensor was determined by immersing the CACQ/ErGO-SPCE in 0.1 M Tris buffer solution containing 0.1 M Zn(II) ion at pH 6.5, and the DPV signal at 0.22 V was recorded every 10 min in 0.1 M KCl containing 10 mM K_3_[Fe(CN)_6_] electron transfer agent.

The linear response range of the Zn(II) sensor was investigated by measuring the voltammetric response of the CACQ/ErGO-modified SPCE with a series of Zn(II) ion solutions in the concentration range of 1 fM to 1 mM at pH 6.5. The interference investigation of the electrochemical Zn(II) ion sensor was then carried out by using a variety of heavy metal ions, such as cadmium(II) [Cd(II)], cobalt(II) [Co(II)], iron(II) [Fe(II)], copper(II) [Cu(II)], and nickel(II) [Ni(II)] ions. The competitive heavy metals were separately prepared in a mixed solution with 1 nM of Zn(II) ion at a 1:1 molar ratio. The discrepancy in the voltammetric response of the Zn(II) ion sensor in the presence and absence of interfering ions was determined in terms of interference percentage. The life span study of the ErGO electrode embedded with CACQ Zn(II) chelating ligand was conducted utilizing sensors from the same production batch, which were prepared on the same day and stored at room temperature in a sealed container for two months. The DPV peak current of the CACQ/ErGO-SPCE electrode sensor towards 0.1 M Zn(II) ion was measured until a significant decline in the DPV response at 0.22 V was observed. The reproducibility of the Zn(II) sensor response was verified via electrochemical assessing several individual sensors with DPV at 1 nM of Zn(II) ion, whilst the repeatability evaluation of the sensor was done by taking several measurements of the same electrode at the same analyte concentration under the same experimental conditions.

### Validation of the voltammetric Zn(II) sensor with standard ICP-MS method

In order to verify the analytical performance of the electrochemical sensor based on the immobilized CACQ Zn(II) chelating ligand on the ErGO-modified SPCE electrode for practical applications, the electrochemical Zn(II) sensor was applied to determine the Zn(II) ion concentration in dietary supplements and statistically compared with the results obtained by ICP-MS standard method using a *t*-test. All the glassware used in the validation study was prepped by submerging them completely in the freshly made 10% (v/v) HNO_3_ for 24 h, followed by rinsing with DIW four times before air dried and used. The Zn(II)-containing dietary supplement was purchased from a local pharmacy and digested following the procedure recommended by [46] with some adjustments. Through open-vessel hotplate digestion, 10 mL of concentrated HNO_3_ was gently added to a 100 mL beaker containing 15 mg of the zinc supplement samples. The solution was then heated at 100°C until it became nearly dry before adding an additional 5 mL of concentrated HNO_3_. This procedure was carried out repeatedly until obtaining a clear solution regardless of color. The clear solution was then cooled and diluted into three different concentrations with 1% HNO_3_ solution. The resultant zinc samples were analyzed by the developed electrochemical Zn(II) ion sensor, and the interpolated results acquired from the calibration curve were compared with the results produced from the Zn(II) analysis by using the industry-recognized ICP-MS technique.

## Results and discussion

### Characterization of the carboxylated 8-carboxamidoquinoline chelating ligand

As the determination of Zn(II) ion will be undertaken in liquid media, the addition of a polar carboxylic acid functional group to the chemical sensing probe would enhance the hydrophilicity level in the system [47]. Thus, the as-synthesized 8-carboxamidoquinoline (8-CQ) with an ester side chain was converted to carboxylic acid (CA) side chain to render the quinoline derivative chelating ligand to possess both hydrophobic and hydrophilic portions in its structure that encouraging chemical stability during metal-ligand interactions, thus raising their binding affinity towards Zn(II) ion [48].

The chemical characteristics of the final product of the as-synthesized CACQ (Yield: 87%) are as follows. The CACQ compound’s melting point was determined to be between 203.5°C and 204.7°C. Given the difference in how long it takes a compound to melt from beginning to end, a modest melting point range of less than 2°C typically implies a fairly pure product is produced [49]. The results of the physical and chemical characterizations of the CACQ ligand compound including ^1^H and ^13^C NMR, FTIR-ATR, and ESI-MS analyses are provided in the Supporting information, i.e. S1 and S2 Appendices. ^1^H NMR signals of CACQ (400 MHz, DMSO-d_6_) were found at δ (ppm): 7.712–7.638 (2H aromatic, m), 7.797–7.774 (J = 1.2, 8.0 Hz, 1 H aromatic, dd), 8.481–8.457 (J = 1.6, 4.0 Hz, 1 H aromatic, dd), 8.675–8.653 (J = 1.2, 7.6 Hz, 1 H aromatic, dd), 8.990–8.975 (J = 1.6, 8.0 Hz, 1 H aromatic, dd), and 11.10 (1 H amide, s). In deuterated protic solvent, e.g. DMSO-d_6_, the hydrogen from labile protons like S-H, N-H, and O-H might infrequently be unviewable due to their high exchange rate between H and D [50]. Thus, OH is absent (S1 Appendix). Meanwhile, ^13^C NMR signals of CACQ (100.6 MHz, DMSO-d_6_) were found at δ (ppm): 117.13 (CH Ar), 122.99 (CH Ar), 124.05 (CH Ar), 127.34 (CH Ar), 128.18 (C Ar), 132.60 (C Ar-NH), 137.33 (CH Ar), 138.07 (C Ar-N), 149.89 (CH Ar-N), 157.05 (C amide), and 162.18(C carboxylic acid). (S1 Appendix).

The FTIR absorption frequencies of the CACQ compound were revealed at the wavenumber of 805 cm^-1^, 756 cm^-1^ (*sp*^2^ C-H bend), 1502 cm^-1^ (ring stretch of C = C-C), 1304 cm^-1^ (C = N stretch), 1690 cm^-1^ (secondary amide group (O = C-NH)), and 3444 cm^-1^ (N-H stretch). The surfacing of strong and broad OH stretching at 3087 cm^-1^ indicates the existence of carboxylic acid (COOH) functional group (S2 Appendix). The calculated molecular weight of CACQ (C_11_H_8_N_2_O_3_) is 216.1928 g mol^-1^. According to the compound’s affinity for proton, the ESI technique can be applied in either a positive (ES+) or negative (ES-) ion mode [51]. In the positive mode of direct ESI-MS infusion, the computed m/z for CACQ was determined to be 217.06 a.m.u. [C_11_H_8_N_2_O_3_ + H]^+^ (S2 Appendix) since protonated molecule [H]^+^ is often used as an ionization method in ESI-MS [52]. These verified the successful synthesis of the CACQ ligand molecule.

### Electrochemical modification of SPCE with graphene sheets and quinoline derivative

**Fig 2:**
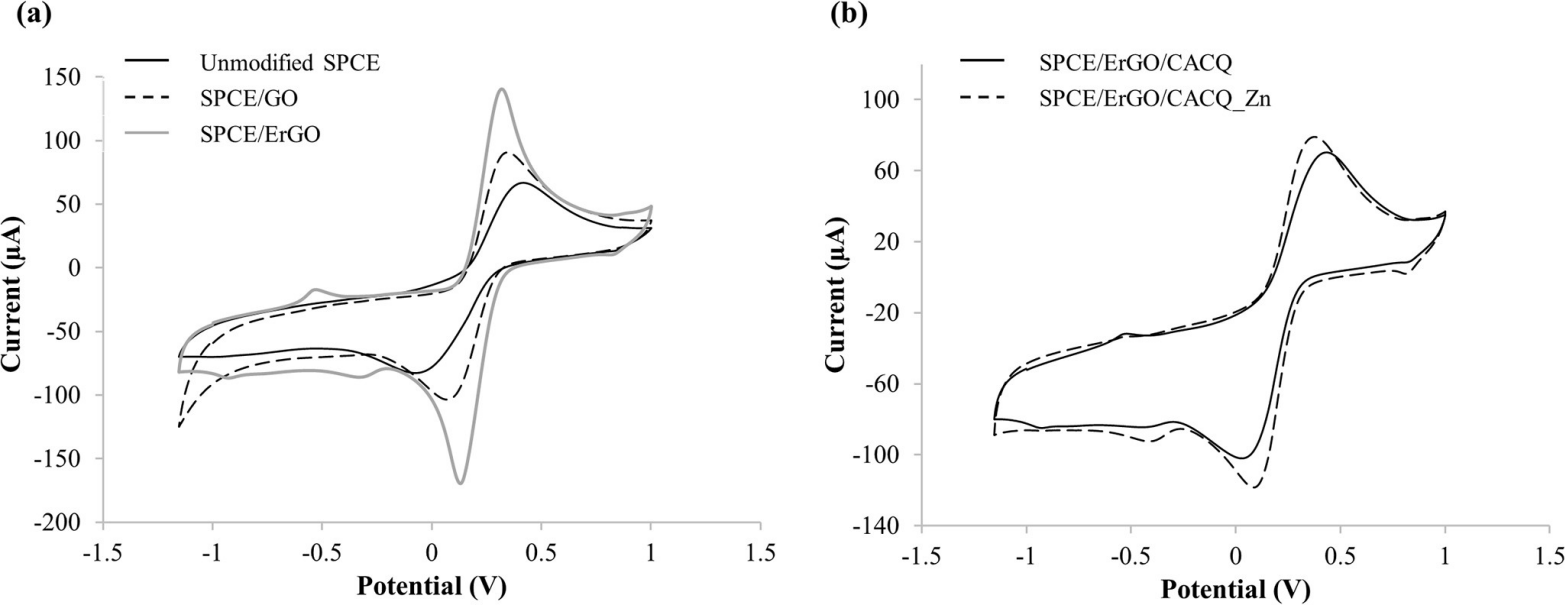
Cyclic voltammograms of sensor. (a) Cyclic voltammograms of bare SPCE, GO-modified SPCE, and ErGO-modified SPCE. (b) Cyclic voltammograms of CACQ ionophore-modified ErGO electrode in the absence and presence of 0.1 M Zn(II) ion. The CV experiments were conducted in 0.1 M KCl containing 10 mM K_3_[Fe(CN)_6_] redox indicator at the scan rate of 100 mV s^-1^ versus Ag/AgCl reference electrode.

An effective and well-liked cyclic voltammetry (CV) is frequently used to study chemical reactions that are initiated by electron transfer and how the molecular species are reduced and oxidized. The CV measurements were conducted stepwise, from the bare SPCE to the fully modified CACQ/ErGO-SPCE including the CV response after the reaction with Zn(II). Each cyclic voltammogram represents the characteristic redox spectrum specific to the corresponding modified electrode. The cyclic voltammogram of unmodified bare SPCE in Fig 2a shows well-defined redox peaks of the K_3_[Fe(CN)_6_] redox indicator at anodic peak potential, *E*_pa_ = 0.4160 V and cathodic peak potential, *E*_pc_ = -0.0693 V. The anodic peak current to cathodic peak current ratio (**|***i*_pa_*/i*_pc_**|**) and peak potential separation (Δ*E*_p_) are the signs of a reversible redox system [53].

It was noted that modification of the SPCE surface with GO enhanced the redox peaks’ current by a factor of 1.3 with a smaller Δ*E*_p_ at 0.2747 V attained, which indicates a faster electron transfer rate at the GO-modified SPCE-solution interface. As the GO electrode underwent a facile and green electrochemical reduction process, the resulting ErGO-modified SPCE revealed a significant heightening in the redox current peaks with an enhancement factor of 2.1, and the smallest Δ*E*_p_ value was acquired at 0.1862 V. The ErGO electrode showed a greater electron transfer rate than the GO electrode due to the recovery of the graphitic network of *sp*^2^ bonds following the electrochemical reduction of some functional groups with negative charges (e.g. -COOH, -COOR) on the GO sheets [54]. Apparently, the differences in oxygen-containing moieties presented within the exfoliation of GO would result in different electrochemical signals by adjusting the duration of the reduction process [55]. The elimination of these functional groups via electrochemical reduction procedure also resulted in the eradication of the repulsive contact between the anionic ferricyanide [[Fe(CN)_6_]^3−^] ion and the ErGO-modified SPCE, thereby permitting the electrons to travel quickly at the ErGO-modified electrode-solution interface. The summary of cyclic voltammetric data recorded at the unmodified electrode and different modified SPCEs in 0.1 M KCl containing 10 mM K_3_[Fe(CN)_6_] is tabulated in Table 1.

**Table 1:** Summary of cyclic voltammetric data recorded at the unmodified electrode and different modified SPCEs in 0.1 M KCl containing 10 mM K_3_[Fe(CN)_6_], including anodic peak potential (*E*_pa_), cathodic peak potential (*E*_pc_), peak separation potential (ΔE_p_), anodic peak current (*i*_pa_), cathodic peak current (*i*_pc_), and anodic-to-cathodic peak current ratio (|*i*_pa_*/i*_pc_|).

Working electrode	*E*_pa_ (mV)	*E*_pc_ (mV)	Δ*E*_p_ (mV)	*i*_pa_ (μA)	*i*_pc_ (μA)	|*i*_pa_*/i*_pc_|
Unmodified SPCE	415.96 ± 2.31	-69.27 ± 0.87	485.23	66.91 ± 0.55	-82.70 ± 0.78	0.81
GO-SPCE	345.76 ± 2.08	71.11 ± 1.10	274.75	90.83 ± 0.24	-103.60 ± 0.19	0.88
ErGO-SPCE	318.30 ± 0.73	132.14 ± 1.29	186.16	140.60 ± 0.85	-169.56 ± 0.35	0.83
CACQ/ErGO-SPCE	431.21 ± 1.31	28.38 ± 0.65	402.80	70.12± 0.21	-102.02± 0.34	0.69
Zn(II)/CACQ/ErGO-SPCE	376.28 ± 1.52	86.36 ± 1.20	289.90	78.92± 0.14	-118.44± 0.16	0.67

The cyclic voltammograms in Fig 2b represent the CACQ ionophore-modified ErGO electrode before and after complexation with Zn(II) ion. Grafting of the quinoline derivative chelating ligand to the ErGO electrode surface by π−π stacking interactions has somehow reduced the redox peak current intensity and enlarged the peak-to-peak separation potential (Table 1). This is mainly attributed to the electrostatic repulsion between the negatively charged carboxyl moieties of the immobilized CACQ molecules and the anionic Fe[(CN)_6_]^3−^ ions at the electrode-electrolyte interface. A remarkable enhancement of redox peak current associated with narrower Δ*E*_p_ was observed upon binding of the Zn(II) ions to the green CACQ/ErGO-modified SPCE via coordination chemistry as a result of the formation of immobilized CACQ-Zn(II) chelate complex, i.e. the charge-transfer complex that produced internal redox process on the ErGO electrode surface. K_3_[Fe(CN)_6_] redox probe was employed as the diffusive electron shuttling agent in order to mediate rapid electron transfer from the metal-chelation reaction to the electrode surface.

Although statistical analysis was not applied to the potential values and current ratios, a qualitative assessment of the data reveals consistent trends across the different samples. The observed consistency in i_pa_/i_pc_ ratios and peak potentials supports the reliability of the measurements.

### Physicochemical and morphological characterization of the modified SPCE

**Fig 3:**
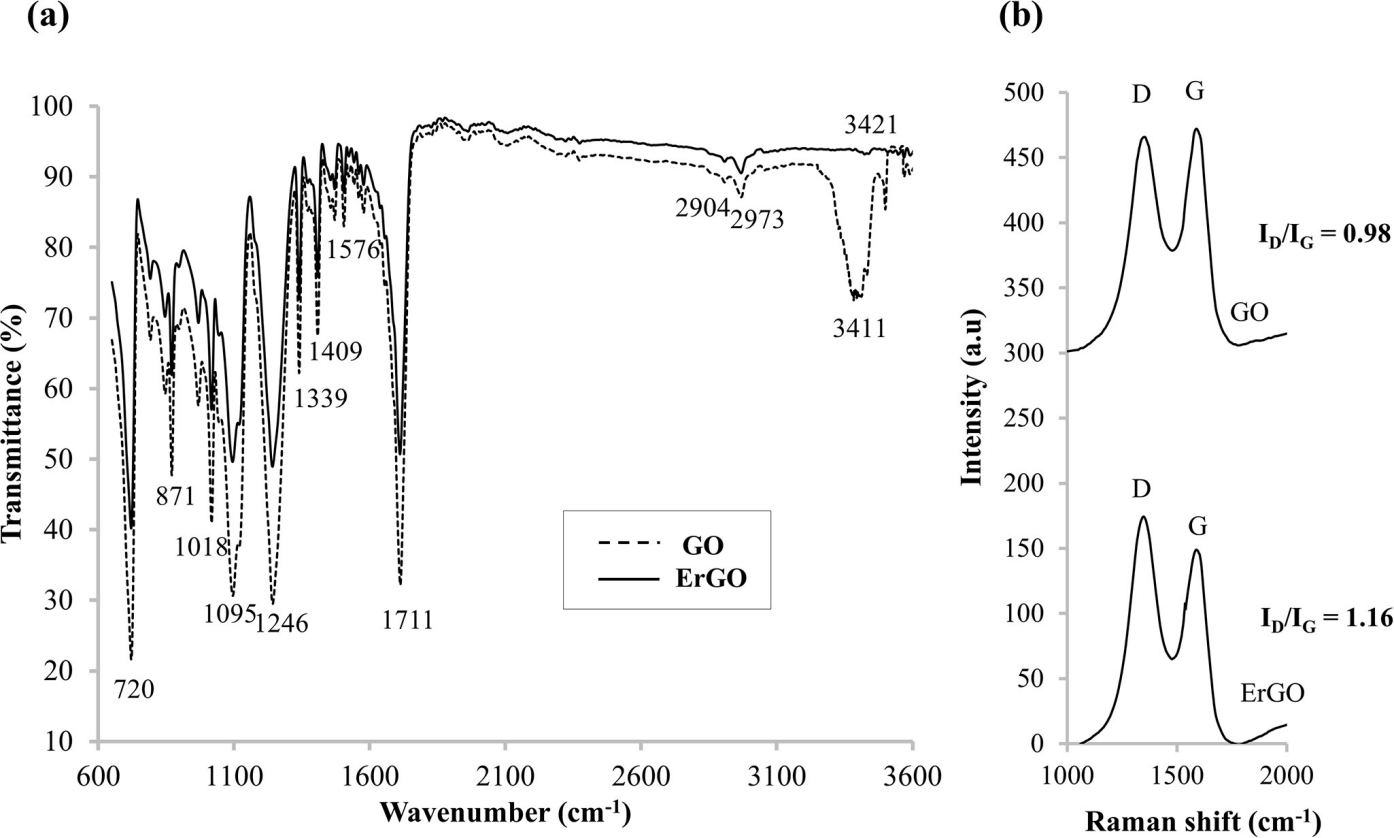
The superimposed spectra. (a) ATR-FTIR spectra and (b) Raman spectra of the as-prepared GO-SPCE and ErGO-SPCE.

The presence of various functional groups, including functional groups containing oxygen in the graphene structures can be characterized with the ATR-FTIR vibrational spectroscopic technique. In Fig 3, the FTIR spectra are displayed in the range of 3600 to 600 cm^−1^. This specific range was chosen to provide a clearer and more detailed view of the significant absorption bands relevant to our study, which are crucial for the analysis and interpretation of our results. The superimposed FTIR spectra of the as-synthesized GO and ErGO is shown in Fig 3a.

The “fingerprint” peaks of GO and ErGO corroborated those of the previously reported studies [56–63]. Carboxylic acids showed a strong and wide band of the O–H stretch at the absorption band of 3411 cm^-1^. The absorption peaks at 2973 cm^-1^ and 2904 cm^-1^ are symbolized symmetric and anti-symmetric stretching vibrations of CH_2_ of the GO [57, 59]. The intense band at the wavenumber of 1711 cm^-1^ is attributable to the carbonyl (C = O) stretch of the carboxyl functional group and in-plane of O-C-H and C-O-H deformations [56, 58, 60]. Absorption at approximately 1600 cm^-1^ is associated with several skeletal vibrations of C = C aromatic of the graphitic structures. Anti-symmetric and symmetric COO^-^ are perceived at the absorption bands of 1409 cm^-1^ and 1339 cm^-1^, respectively [58]. The absorption peak at 1246 cm^-1^ corresponds to the C-OH stretch of the alcohol group [61]; 1099 cm^-1^ is attributed to the C-O stretching vibration of C-O-C [56]; and 1018 cm^-1^ represents the stretching vibration of C-O and C-C [62]. Aromatic C-H out-of-plane bending vibrations are in the range between 875 cm^-1^ and 750 cm^-1^ [62]. Absorption peaks for epoxides stretch are found at 871 cm^-1^ (asymmetric C-O-C stretch) and 720 cm^-1^ (symmetric C-O-C stretch) [63].

As can be observed from Fig 3a, the FTIR spectrum for ErGO shows a considerable reduction in the intensity of the absorption bands, which are related to the oxygen-containing functional groups in comparison to the absorption peaks’ intensities of GO. This signifies that the graphite had successfully been oxidized into GO, followed by electroreduction into reduced GO. The deoxygenation of the carboxyl rendered stretching vibrations of the O-H groups at 3411 cm^-1^ of GO to almost vanish at 3421 cm^-1^ for ErGO. The stretching vibration of CH_2_ at 2904 cm^-1^ of ErGO was still visible after the electrochemical reduction of GO, indicating the presence of C-H groups. The FTIR absorption peak at 1576 cm^-1^ is caused by the graphitic domains, confirming the formation of the *sp*^2^ carbon structure of ErGO. Because most of the functional groups containing oxygen in the graphene structure had not completely vanished, suggesting that the GO was not entirely reduced via electrochemical reduction, and the presence of some oxygen-functional groups, e.g. ketone carbonyls, epoxides, alcohols, and carboxylic groups still exist in the finale ErGO.

Further, the highly effective non-destructive technique of Raman spectroscopy was used to examine the electronic structures of the modified carbonaceous electrodes [64]. For both GO and ErGO, which were both deposited on the SPCE, the D and G Raman bands were found at 1365 cm^-1^ and 1596 cm^-1^ (GO), and 1359 cm^-1^, and 1604 cm^-1^ (ErGO), respectively. Fig 3b points to the D/G Raman intensity ratio for the ErGO (1.18) being higher compared to GO (0.98) as a result of electrochemical reduction altering the GO structure [65–67], which led to the elimination of functional groups and the emergence of in-plane *sp*^3^ and *sp*^2^ hybridizations [68–70].

**Fig 4:**
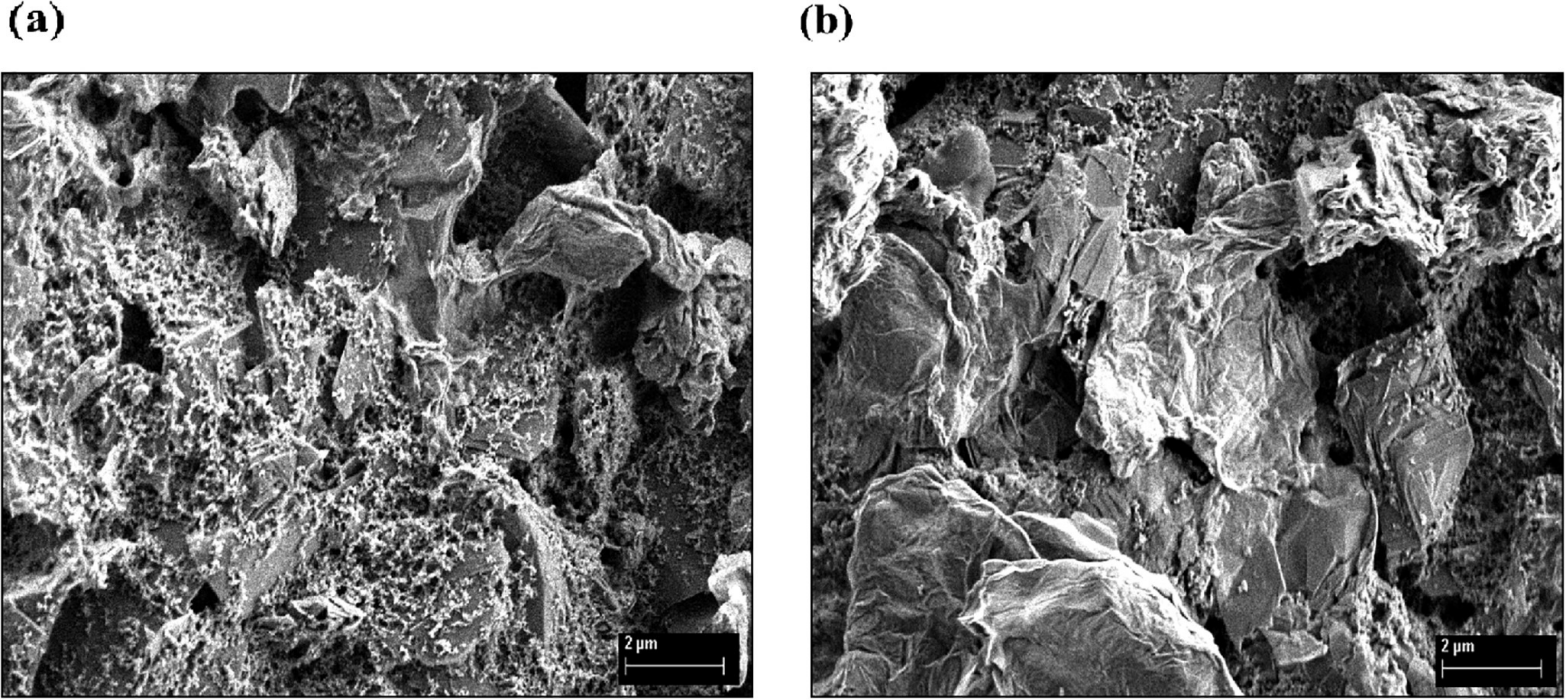
Surface morphologies of materials. (a) GO-SPCE and (b) ErGO-SPCE. The FESEM images were taken at a magnification of 5.0 k× and an accelerating voltage of 3 kV.

Analysis of surface morphologies by field-emission scanning electron microscopy (FESEM) of modified electrodes including GO-SPCE and ErGO-SPCE are demonstrated in Fig 4. The pristine GO is seen in Fig 4a as crumpled structures and sheet-like shapes [71]. The electro-reduced GO nanosheets are exhibited in Fig 4b with flake-like forms and wrinkly surface morphology. The layered ErGO sheets were also closely packed, with the margin of each layer being distinct from the crumpled portions. The ErGO lamellae sheet with a rough surface structure increased the surface area, which is essential in providing ample binding sites for the chemical receptors to be immobilized on the electrode surface [72]. The CACQ ionophore covering the surface area of the wrinkled ErGO electrode might be a result of the π−π stacking bonding interactions. The CACQ-modified wrinkled ErGO-SPCE afforded a large surface-to-volume ratio with multiple binding sites for multiple dative covalent binding with Zn(II) ion. The term ‘wrinkled’ is employed to portray the observed morphology of the electrode surfaces, depicted by noticeable folds and undulations. These features are apparent in the FESEM images, where the surface occurs to have a wrinkled texture.

Energy dispersive X-ray spectroscopy (EDX) mapping was then used to analyze the elements on each modified electrode surface. The components, such as carbon (C), oxygen (O), and zinc (Zn) were observed to change according to weight percentage (wt%) and the color mapping for each electrode indicates the presence of each extended layer being immobilized on the SPCE surface (S2 Fig). The carbon-oxygen ratio of ErGO-SPCE (12.19) (S2c Fig) was noticed to be higher compared to that of the GO-SPCE electrode (5.012) (S2b Fig) due to the removal of oxygen-containing functional groups through electrochemical reduction of the GO-modified SPCE. In view of the presence of amide and carboxylic acid functional groups in the CACQ chelating agent, it enhanced the wt% of the O component (green color) for the CACQ-modified ErGO electrode (S2d Fig). The presence of Zn element in S2e Fig affirms that the dative covalent binding events between CACQ/ErGO-SPCE and Zn(II) ions had been taken place.

### Optimizing parameters influencing the DPV response of the Zn(II) sensor based on ErGO supporting matrix

**Fig 5:**
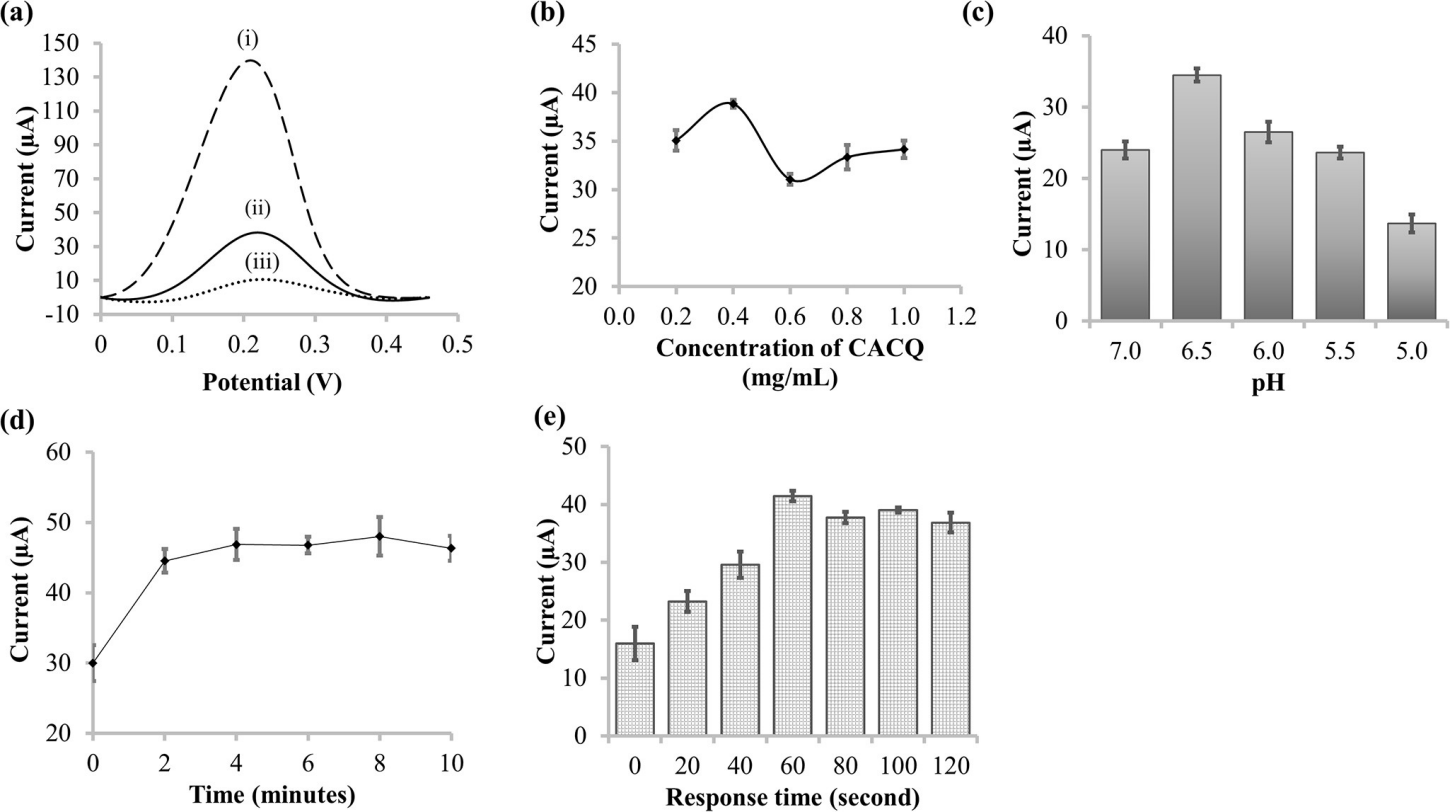
DPV response for optimization of Zn(II) sensor. (a) DPV response of the (i) SPCE-ErGO, (ii) Zn(II)/CACQ/ErGO-SPCE, and (iii) CACQ/ErGO-SPCE in 0.1 M KCl containing 10 mM K_3_[Fe(CN)_6_] in the potential range of 0.0 V to +0.5 V versus Ag/AgCl reference electrode; (b) the effect of CACQ organic ligand concentration from 0.2–1.0 mg mL^-1^ on the DPV response of the Zn(II) sensor at 0.22 V; (c) The effect of pH on the Zn(II) sensor response by using 0.1 M Tris buffer solution with pH adjusted between pH 5.0 and pH 7.0; (d) leaching test profile of the Zn(II) sensor based on quinoline derivative chelating ligand-modified ErGO electrode; and (e) response time trending of the Zn(II) sensor towards DPV detection of 0.1 M Zn(II) ion at 0.22 V.

The differential pulse voltammograms obtained for (i) ErGO-SPCE, (ii) Zn(II)/CACQ/ErGO-SPCE, and (iii) CACQ/ErGO-SPCE in 0.1 M KCl containing 10 mM [K_3_[Fe(CN)_6_] electron shuttling agent within the potential window of 0.0 V to +0.5 V versus Ag/AgCl reference electrode (Fig 5a) strongly corroborated the CV findings, providing further evidence of the formation of immobilized CACQ-Zn(II) chelate via coordinate covalent linkage on the ErGO electrode surface, and the maximum DPV peak potential at 0.22 V was determined to be the operational potential for voltammetric detection of Zn(II) ion.

The electrochemical Zn(II) sensor based on the immobilized 8-carboxamidoquinoline receptor, was initially optimized for CACQ loading between 0.2 mg mL^-1^ and 1.0 mg mL^-1^ towards the detection of 0.1 M Zn(II) ion. The effect of CACQ probe loading variations on the DPV response of the Zn(II) sensor in Fig 5b shows that the concentration of CACQ compound of 0.4 mg mL^-1^ gave a maximum DPV response at 0.22 V based on the variation in DPV response before and after the electrochemical sensor reacted with 0.1 M Zn(II) ion. The observed curvature of the current versus concentration curve in Fig 5b, showing a peak at 0.4 mg/mL CACQ, a dip at 0.6 mg/mL, and a slight increase up to 1.0 mg/mL leading to an almost plateau, can be explained by the concentration-dependent interaction of CACQ molecules with the electrode surface and the behavior of excess unbound CACQ in the Zn(II) ion solution, along with metal chelation dynamics between CACQ and Zn(II).

Initially, as the CACQ concentration increases, the current also rises due to the increased availability of CACQ molecules to bind with Zn(II). This binding process may enhance the electrochemical signal as the CACQ-Zn(II) complex formation proceeds. At lower concentrations (up to 0.4 mg/mL), CACQ molecules likely adsorb uniformly and efficiently onto the electrode, facilitating optimal electron transfer and resulting in a maximum current. At this concentration, the system may reach a saturation point where the maximum number of Zn(II) ions are complexed with CACQ, leading to the observed peak in current. This suggests a high degree of interaction or binding affinity between CACQ and Zn(II), resulting in a maximal electrochemical response.

As the concentration increases to 0.6 mg/mL, the electrode surface may become saturated or crowded, leading to a reorganization of the adsorbed molecules on the surface. This reorganization could reduce the availability of active sites for Zn(II) binding, resulting in a dip in the current. Similar surface saturation effects have been observed in various electrochemical systems, where the excessive presence of binding molecules can disrupt the optimal electrochemical signal [73]. Additionally, excess CACQ might begin to aggregate, further reducing the number of electroactive sites and thus causing the observed drop in current. Also, the presence of unbound CACQ in the solution, due to its carboxylic acid (CA) side chain with both hydrophobic and hydrophilic portions, may lead to partial aggregation or interactions that reduce available electroactive sites. There is also a possibility that the excess unbound CACQ may remain in the solution and form CACQ-Zn complexes during immersion in the Zn(II) ion solution. This could potentially affect the overall binding dynamics and the electrochemical response, as the unbound CACQ molecules could interact with Zn(II) ions in the solution rather than at the electrode surface, leading to the formation of these complexes in the bulk solution. This behavior might contribute to the observed changes in current, especially at higher concentrations where the availability of free CACQ in the solution increases.

The slight increase in current at 1.0 mg/mL could be due to additional binding interactions or reorganization of CACQ-Zn(II) complexes, partially restoring electron transfer efficiency. However, the current does not return to the peak observed at 0.4 mg/mL, likely due to complex interactions between the excess ligand, Zn(II) ions, and the electrode surface. These complex interactions might hinder the optimal alignment for efficient electron transfer, preventing the current from returning to its earlier maximum. This behavior emphasizes the complexity of surface interactions, where increasing concentrations of the probe beyond optimal levels can lead to decreased sensor performance due to molecular crowding and complexation effects [74].

The effect of 0.1 M Tris buffer solution pH on the electrochemical Zn(II) ion sensor response was then performed by using the CACQ optimum loading at 0.4 mg mL^-1^ between pH 5.0 and pH 7.0 against the detection of 0.1 M Zn(II) ion. This is because pH has a significant impact on the stability and production of chelates where metals frequently form insoluble hydroxides in basic circumstances, which makes them less susceptible to chelating agents, and most chelating agents are problematic in highly acidic. It appeared that Zn(II) ion bound most effectively to the chelating ligand at pH 6.5, as indicated by the optimal DPV response in Fig 5c. This observation is consistent with the well-established principles of Zn(II) coordination chemistry, where pH plays a critical role in influencing the ligand environment and binding efficiency. Notably, the highest response at pH 6.5 aligns with previous research, which observed a similar trend during the examination on the impact of aqueous medium pH on Zn(II) fluorescence sensor response under neutral conditions [75].

Next, a leaching test was performed by submerging the CACQ/ErGO-SPCE electrode in DIW before dipping it in an analyte of 0.1 M Zn(II) ion at pH 6.5. The DPV response of the Zn(II) sensor at 0.22 V increased to a stable current after two minutes of electrode immersion time in DIW, and become constant thereafter as shown by the line graph of the DPV Zn(II) sensor response as a function of sensor immersion duration in Fig 5d. The system was unstable in the first two minutes was due to the discharging of excess CACQ ionophore into the water phase, which became a barrier to the electron transfer at the electrode-electrolyte interface. A constant DPV response at 0.22 V was observed from two minutes of the electrode immersion time and onwards suggesting that there was no leaching of CACQ compound occurred, and a strong non-covalent attractive force of aromatic-aromatic stacking between the CACQ reagent molecules and the supporting matrix of ErGO material was formed. By submerging the Zn(II) sensor in 0.1 M Zn(II) ion in 0.1 M Tris buffer saline at pH 6.5 from 0–120 s, an optimum response time of the voltammetric Zn(II) sensor was obtained at one minute (Fig 5e), which implies that the electrode surface has reached its maximal Zn(II)-chelation reaction.

### Dynamic linear range, interference effect, reproducibility, repeatability, and long-term stability performances of voltammetric Zn(II) sensor

**Fig 6:**
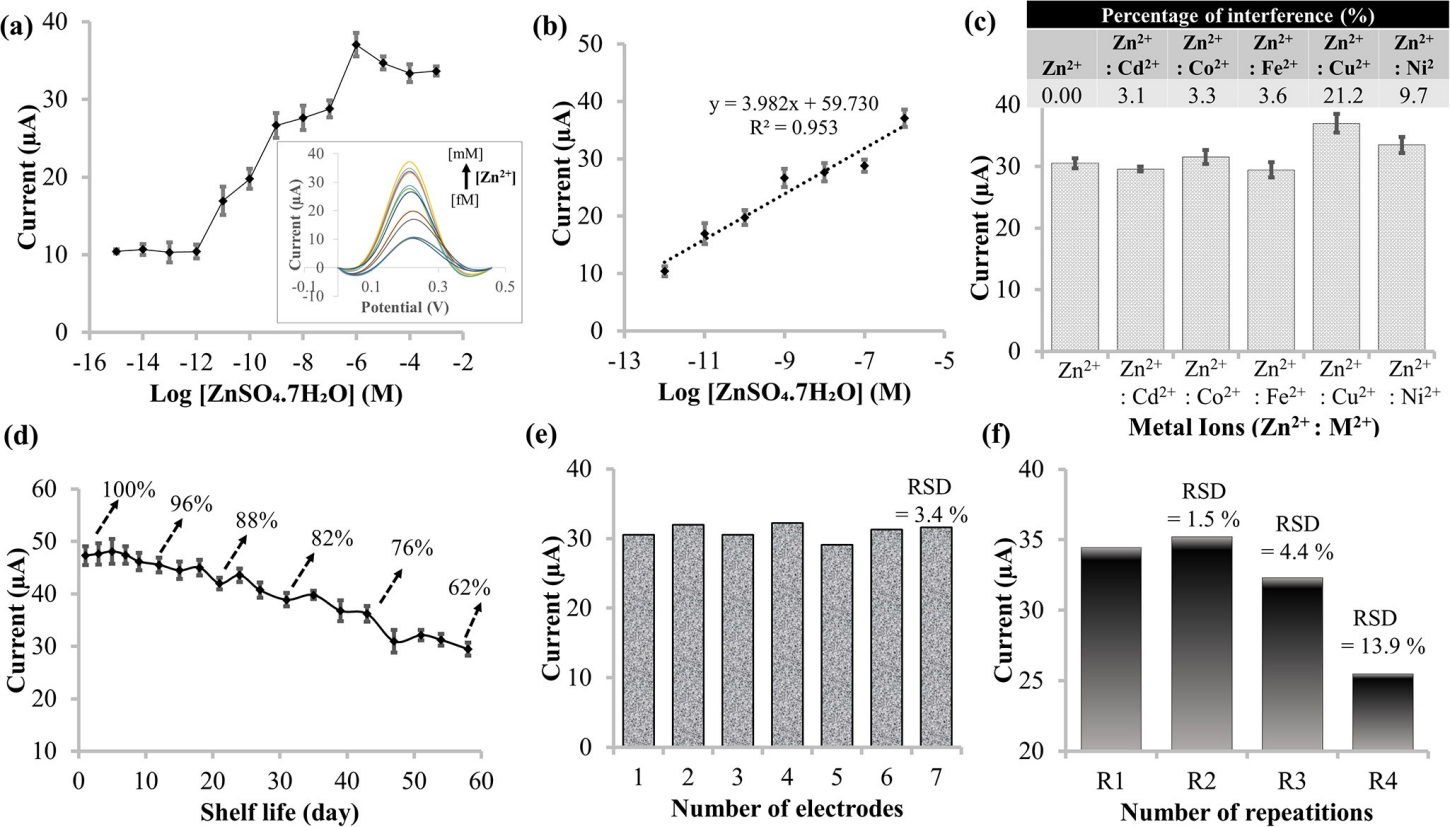
DPV response for performances of voltammetric Zn(II) sensor. (a) Electrochemical response of the CACQ ligand-based Zn(II) sensor over a series of Zn(II) ion concentrations from 1 fM to 1 mM. The inset shows the corresponding voltammetric response of the Zn(II) sensor towards various Zn(II) concentrations; (b) the dynamic linear range of the Zn(II) sensor between 1 pM and 1 μM; (c) The interference effect of the Zn(II) sensor was based on 1:1 molar ratio between Zn(II) ion and interfering ions; (d) the DPV Zn(II) sensor’s shelf-life trending at 0.22 V for 60 days; (e) reproducibility study using seven individual modified-SPCEs for electrochemical quantification of 1 nM Zn(II) ion; and f repeatability study using the same CACQ/ErGO-SPCE electrode for four consecutive analyses of Zn(II) ion under the same investigational conditions.

The DPV response of the CACQ ligand-based Zn(II) sensor over electrochemical detection of Zn(II) ions in the concentration range of 1 fM to 1 mM is demonstrated in Fig 6a, and its corresponding differential pulse voltammograms of the Zn(II) ion sensor response is shown in the inset. The metal-chelation reaction rate increased along with the increase in analyte concentration prompting the K_3_[Fe(CN)_6_] redox probe to diffuse at a greater rate speeding up the redox reaction at the electrode-electrolyte interface.

The DPV current of the Zn(II) sensor rises proportionally with Zn(II) ion concentration from 1 pM to 1 μM Zn(II) ion, attaining a dynamic linear trendline with the linear equation y = 3.9816 x+59.73 (R^2^ = 0.9529) (Fig 6b). The highly sensitive detection of Zn(II) ion down to pM levels was most probably attributed to the CACQ-modified wrinkled ErGO electrode featuring a large surface-to-volume ratio and that serving multiple binding sites for receptor probes, resulting in greater opportunity for multiple dative covalent binding events with Zn(II) via coordination chemistry, and significantly enhanced the electron transfer rate at the electrode surface. The lowest concentration that the proposed Zn(II) electrochemical sensor can detect is referred to as the limit of detection (LOD), which was obtained at 0.53 pM based on the equation LOD = m+3SD, where ‘m’ denotes the average concentration of blank and ‘SD’ denotes the standard deviation of the blank.

Quantitative analysis of the selectivity of the Zn(II) electrochemical sensor was investigated using a variety of metal ions, including Cd(II), Co(II), Fe(II), Cu(II), and Ni(II) ions, and the experimental results appear in Fig 6c. The DPV Zn(II) sensor maintained stable response currents in the presence of all potential interfering ions. The electrochemical sensor’s interference performance was evaluated by determining the percentage of the response current toward the interfering ions to that toward the Zn(II) ion. With the addition of the respective competitive heavy metals at a 1:1 molar ratio between Zn(II) and potential interfering ions, the Zn(II) sensor showed negligible interference effect with an interference percentage of less than 5%. The interference effect from the 1:1 mixture of Zn(II) and Cu(II) ions and mixture of Zn(II) and Ni(II) ions were noticed to be higher than 5% (i.e. 21.2% and 9.7%), probably ascribed to the immobilized CACQ probe, which appeared as a borderline base and has the propensity to create a coordinate covalent binding with borderline acids, such as Zn(II), Cu(II) and Ni(II) ions [76]. This relates to the qualitative explanation of hard and soft acid-base (HSAB), which postulates as hard acids favor hard bases and soft acids favor soft bases that reinforce and stabilize the linkages between the donor and the acceptor [77].

Throughout the 60-day study period of the sensor life span, Fig 6d illustrates the long-term stability of the ErGO electrode embedded with CACQ Zn(II) chelating ligand upon electroanalytical assessment with 0.1 M Zn(II) ion under optimized conditions. The sensor response was found stable up to 5 days of storage period with sensor response retained at 92% compared to its response on the first day. After 20 days of storage, the sensor response gradually decreased to ~88% on the 21^st^ day and ~ 80% on the 39^th^ day. The Zn(II) sensor response continued to decrease with storage time until almost half of its original response on day 58. The limitation of sustainable sensing performance of the CACQ/ErGO-modified electrode might be the inadequate storage conditions that have tampered with the sensor shelf-life. Nonetheless, more than 80% of the sensor’s initial DPV response was retained on the 35^th^ day of storage duration, demonstrating stable adsorption of the heterocyclic CACQ sensing ionophore on the graphene sheets-modified SPCE via non-covalent π–π stacking interaction.

Reproducibility testing of seven repeated experiments over different individual modified-SPCE electrodes afforded relative standard deviation (RSD) values of 3.4%, which substantiates the ability of the Zn(II) electrochemical sensor to generate identical voltammetric responses for a duplicated experimental set-up (Fig 6e). A repeatability study conducted using the same electrochemical sensor throughout four repetitive (*n* = 4) DPV analyses of 1 nM Zn(II) ion obtained satisfactory RSDs of 1.5% (*n* = 2) and 4.4% (*n* = 3) (Fig 6f). The Zn(II) sensor was regenerated with 0.1 M HCl between Zn(II) ion analyses in order to regenerate the chelating sites, whereby the immobilized Zn(II) ions dissociated from the immobilized CACQ-Zn(II) chelate complexes at strong acidic medium and leaving behind the CACQ-modified ErGO electrode, such that it could be reused to re-coordinate with Zn(II) ion in the subsequent experiment. Poor repeatability RSD obtained at 13.9% on the 4^th^ cycle of Zn(II) ion analysis might be due to leaching of the CACQ ionophore from the ErGO electrode as a result of regeneration of the sensor electrode that was performed consecutively by using acidic regeneration solution.

### Validation of voltammetric sensor with ICP-MS for Zn(II) detection in dietary supplements

The tabulation of the validation results obtained with both the electrochemical sensor and ICP-MS methods for the analysis of Zn(II) contents in the dietary supplement samples are outlined in Table 2. The voltammetric sensor responses recorded in terms of DPV peak current were used to estimate the corresponding Zn(II) ion concentration in the digested zinc supplement samples via interpolation analysis. A *t*-test was performed to statistically validate the Zn(II) amounts identified by both the electrochemical sensor and ICP-MS methods at a 95% confidence level (α = 0.05) with four degrees of freedom. The calculated *t*-scores for three samples were found smaller than the critical threshold at *t*_4_ = 2.776 suggesting that no statistically significant difference in the quantitative determination of Zn(II) ion concentration for both approaches. The correlation coefficient (R^2^) between the DPV Zn(II) sensor and ICP-MS methods, which was obtained at a value of near ±1, i.e. 0.9903 (S1 Fig) is another statistical measure showing a perfect correlation between the two methods for detection of Zn(II) ion concentration. Hence, the proposed Zn(II) sensor based on the electrochemical modification of SPCE surface with ErGO integrated with CACQ sensing ionophore can potentially be used for sensitive and reliable assay of Zn(II) content in water, food, pharmaceutical, and biological samples in a simple, facile and economical way.

**Table 2:** The zinc contents in dietary supplements detected by the electrochemical Zn(II) sensor and statistically compared using a *t*-test with the results obtained by ICP-MS standard method.

Sample	DPV sensor current (μA)	Zn(II) ion concentration determined by the electrochemical sensor (nM)	Zn(II) ion concentration determined by ICP-MS (nM)	Relative error between the two methods (%)	*t-test
N1	31.2±0.3	69.2±10.2	73.5±5.0	5.8	0.65
N2	31.8±0.2	99.6±9.8	89.2±10.8	11.7	1.24
N3	32.5±0.2	143.8±18.5	131.9±6.3	8.9	1.04

*The *t* critical value at a confidence level of 95% (α = 0.05) with four degrees of freedom = 2.776.

### Comparison of the performance of the proposed electrode with other modified carbon electrodes for the detection of Zn(II) ion

Table 3 compares the previously reported electroanalytical sensors for Zn(II) ion determination with the proposed electrochemical sensor developed based on an environmentally friendly modified SPCE of electrochemically reduced graphene oxide embedded with CACQ quinoline derivative chelating ligand of Zn(II) in terms of electrochemical technique used, modified working electrodes, linear detection range, the limit of detection, response time, and real sample analysis involved.

**Table 3:** Comparison of the proposed modified-SPCE electrode with ErGO embedded with CACQ chelating agent with previously reported modified electrodes for electrochemical sensing of Zn(II) ion.

Method	Modified electrode	Response time (s)	Linear range (M)	LOD (M)	Real sample	References
^a^ ASV	Screen-printed gold electrodes	90	1.53×10^−6^–1.07×10^−4^	3.82×10^−8^	Seawater sample	[78]
^b^ SW-ASV	modified bidentate Schiff base-membrane/SPCE	60	1.00×10^−6^–1.00×10^−4^	4.6×10^−8^	Blood and urine	[79]
^c^ SWV	Graphite/Carbon Paste with 2-Hydroxy-1,4-naphthoquinone	-	0.47×10^−6^–93.8 ×10^−6^	0.28 ×10^−6^	River waters	[80]
^b^ SW-ASV	Thin gold electrodes sputtered onto nanoporous poly(acrylic acid)-grafted-poly(vinylidene difluoride) (PAA-g-PVDF)	150	1.53×10^−7^–7.65 ×10^−6^	6.42 ×10^−8^	Oil-polluted seawater	[81]
^a^ ASV	Bi/GO-GCE	480	3.06×10^−6^–1.22×10^−4^	9.17×10^−8^	Human seminal fluid	[73]
^d^ DPV	PSS/W-rGO-GCE	300	5.00×10^−9^–7.20×10^−7^	1.70×10^−9^	Seawater	[82]
^d^ DPV	f-MWCNTs/CS/PB/AuE	5	4.13×10^−6^–1.07×10^−4^	2.6×10^−7^	Drinking water	[83]
^e^ LSV	L-SPCE	10	1.00×10^−6^–1.00×10^−1^	3.5×10^−6^	Pharmaceutical tablets	[84]
^d^ DPV	CACQ/ErGO-SPCE	60	1.00×10^−12^–1.00×10^−6^	5.30×10^−13^	Dietary supplements	This work

^a^ ASV: Anodic stripping voltammetry

^b^ SW-ASV: Square-wave anodic stripping voltammetry

^c^ SWV: Square wave voltammetry

^d^ DPV: Differential pulse voltammetry

^e^ LSV: Linear sweep voltammetry

It is noted that the proposed green CACQ-modified wrinkled ErGO electrode obtained better electroanalytical performance for Zn(II) ion via DPV compared to a flow electrochemical analyses of Zn(II) by stripping voltammetry on graphite felt electrode [79]; graphite/carbon paste electrode modified with 2-Hydroxy-1,4-naphthoquinone via square wave voltammetry (SWV) [80]; 3-D printed carbon nanofiber–graphite–polystyrene electrode for anodic stripping voltammetric determination of Zn(II) [81]; bismuth (Bi) and graphene oxide GO modified glassy carbon electrode (GCE) by anodic stripping voltammetry (ASV) [73]; and poly(sodium 4-styrenesulfonate)/rGO composite-modified GCE via DPV [82]. The developed CACQ/ErGO-SPCE demonstrated rapid detection time, higher sensitivity, and a wider linear Zn(II) ion concentration range due to the flake-like forms and wrinkly surface morphology of the layered electro-reduced GO nanosheets, which have considerably increased the surface area to promote multiple binding of the chelate complexes and enhanced the electron transfer rate at the electrode surface.

## Conclusions

A simple and environmentally friendly electrochemical reduction of GO onto SPCE with the immobilization of the ligand 8-carboxamidoquinoline, CACQ, has demonstrated high sensitivity electrochemical detection of Zn(II) ion by DPV, likely because the CACQ/ErGO-modified electrode possessed a strong π-electronic configuration that led to better conductivity, less hydrophilic, and high surface area-to-volume ratio that allowed high loading of ligand molecules for binding with Zn(II) ions. Despite using a disposable SPCE electrode, this sensor demonstrated analytical properties that were comparable to or better than those of other investigations in the literature as well as corresponded perfectly with the standard ICP-MS method for detection of Zn(II) ion concentration in digested zinc supplement samples. In light of this, the voltammetric Zn(II) sensor developed in this study has considerable potential for determining Zn(II) ions at low concentrations in various sample matrices e.g. food, biological samples and pharmaceutical products.

## Supporting information

S1 Appendix ^1^H NMR and 13C NMR of CACQ. (I) ^1^H NMR spectrum of CACQ, (II) List of the proton’s signal of CACQ, specifying the chemical shift (δ), the multiplicity, the integration, the coupling constants (in Hz), the type of proton and assignation of protons, (III) 13C NMR spectrum of CACQ, and (IV) List of the carbon’s signal of CACQ, specifying the chemical shift (δ), and assignation of carbons.
(DOCX)

S2 Appendix ATR-FTIR and ESI-MS of CACQ. (I) ATR-FTIR spectrum of CACQ, (II) ATR-FTIR absorption frequencies of CACQ, and (III) ESI-MS spectrum of CACQ [C_11_H_8_N_2_O_3_ + H]^+^. Positive mode.
(DOCX)

S1 Fig Correlation between the two methods for detection of Zn(II) ion concentration. ICP-MS versus proposed sensor.
(TIF)

S2 Fig Elemental analysis of fabricated SPCE by FESEM-EDX. Elemental analysis of (a) unmodified SPCE and modified SPCEs, i.e. (b) GO-SPCE, (c) ErGO-SPCE, (d) CACQ/ErGO-SPCE, and (e) Zn(II)/CACQ/ErGO-SPCE. The EDX mapping’s color representations: red for carbon, green for oxygen, and orange for zinc.
(TIF)

S3 Fig Graphical abstract. (TIF)
