# Supplementary material for: Electrochemically reduced graphene oxide integrated with carboxylated-8-carboxamidoquinoline: A platform for highly sensitive voltammetric detection of Zn(II) ion by screen-printed carbon electrode
Source: PLoS One. 2025 Feb 7;20(2):e0315974. doi: 10.1371/journal.pone.0315974 (PMC11805387; doi:10.1371/journal.pone.0315974)
Supplement: S1 Appendix — (I) 1H NMR spectrum of CACQ, (II) List of the proton’s signal of CACQ, specifying the chemical shift (δ), the multiplicity, the integration, the coupling constants (in Hz), the type of proton and assignation of protons, (III) 13C NMR spectrum of CACQ, and (IV) List of the carbon’s signal of CACQ, specifying the chemical shift (δ), and assignation of carbons. (DOCX) [file pone.0315974.s001.docx]

**Supporting Information**

**Electrochemically reduced graphene oxide integrated with carboxylated-8-carboxamidoquinoline: A platform for highly sensitive voltammetric detection of Zn(II) ion by screen-printed carbon electrode**

Nur Syamimi Mohamad^1^, Nurul Izzaty Hassan^2^, Choo Ta Goh^1^, Ling Ling Tan^1,^*

**1** Southeast Asia Disaster Prevention Research Initiative (SEADPRI), Institute for Environment and Development (LESTARI), Universiti Kebangsaan Malaysia, 43600 UKM Bangi, Selangor Darul Ehsan, Malaysia

**2** Department of Chemical Sciences, Faculty of Science and Technology, Universiti Kebangsaan Malaysia, 43600 UKM Bangi, Selangor Darul Ehsan, Malaysia

*Author to whom correspondence should be addressed: Ling Ling Tan

E-mail address: lingling@ukm.edu.my

Table of Contents

1. ^1^H NMR spectrum of CACQ …………………………………….……...……...1-2
2. List of the proton’s signal of CACQ, specifying the chemical shift (δ), the multiplicity, the integration, the coupling constants (in Hz), the type of proton and assignation of protons.……………………...………………………………...…...3
3. ^13^C NMR spectrum of CACQ……………………………………...……..........4-5
4. List of the carbon’s signal of CACQ, specifying the chemical shift (δ), and assignation of carbons …………………………………………………………….6

^1^H NMR spectrum of CACQ. NMR spectra were recorded on a NMR spectrometer (Advance 400 III HD Bruker) at a magnetic field strength of 400.17 MHz. Measurements were performed at 22.85°C using deuterated DMSO as the solvent, with TMS, Si(CH_3_)_4_ as the internal reference. The sample concentration was 1 mM, and solvent signal suppression was applied where necessary. Detailed chemical shift (δ), the multiplicity, the integration, the coupling constants (in Hz), the type of proton and assignation of protons are provided.


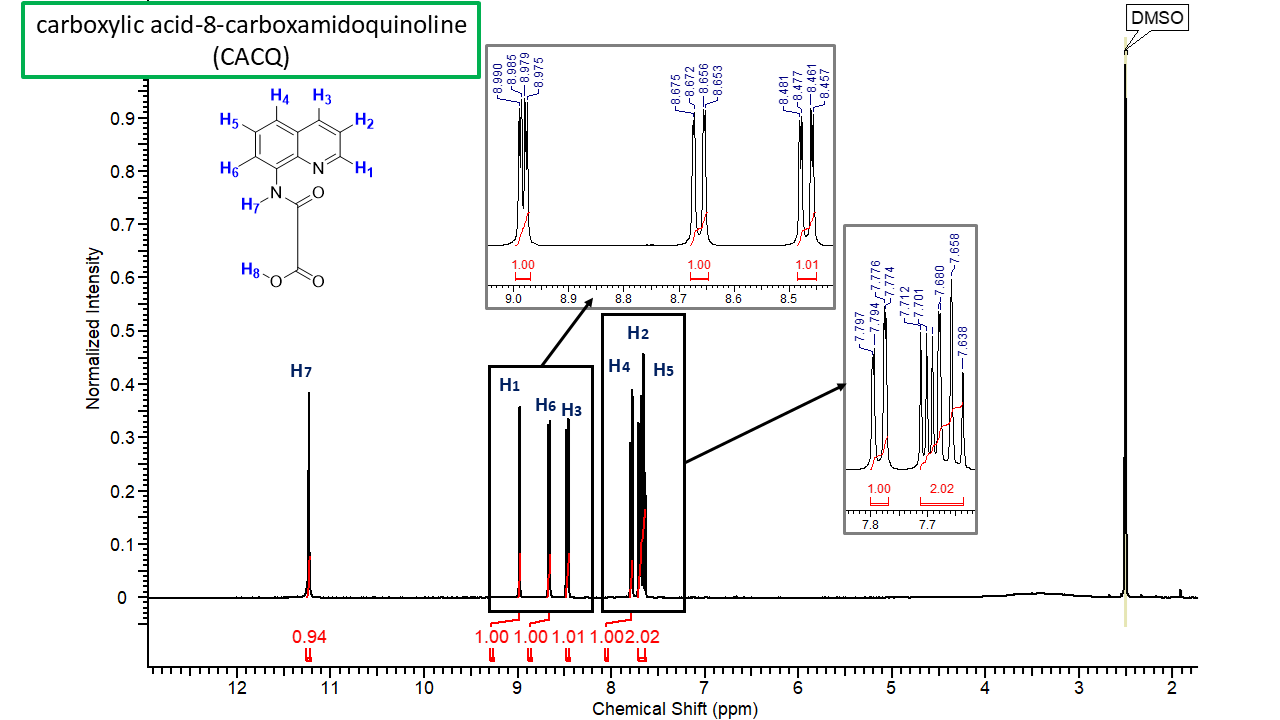


List of the proton’s signal of CACQ, specifying the chemical shift (δ), the multiplicity, the integration, the coupling constants (in Hz), the type of proton and assignation of protons.

| **Chemical shift (δ)** | **Multiplicity** | **Integration** | **Coupling constants (In Hz)** | **Type of proton** | **Assignation** |
| --- | --- | --- | --- | --- | --- |
| 11.10 | singlet | 1 | - | N-H | 7 |
| 8.990-8.975 | Doublet of doublets | 1 | 1.6, 8.0 | Ar-H | 6 |
| 8.675-8.653 | Doublet of doublets | 1 | 1.2, 7.6 | Ar-H | 3 |
| 8.481-8.457 | Doublet of doublets | 1 | 1.6, 4.0 | Ar-H | 4 |
| 7.797-7.774 | Doublet of doublets | 1 | 1.2, 8.0 | Ar-H | 1 |
| 7.712-7.638 | Multiplets | 2 | - | Ar-H | 2 and 5 |

^13^C NMR spectrum of CACQ. NMR spectra were recorded on an NMR spectrometer (Advance 400 III HD Bruker) at a magnetic field strength of 100.63 MHz. Measurements were performed at 22.85°C using deuterated DMSO as the solvent, with TMS, Si(CH_3_)_4_ as the internal reference. The sample concentration was 1 mM, and solvent signal suppression was applied where necessary. Detailed chemical shifts (δ), and assignation of carbons are provided.


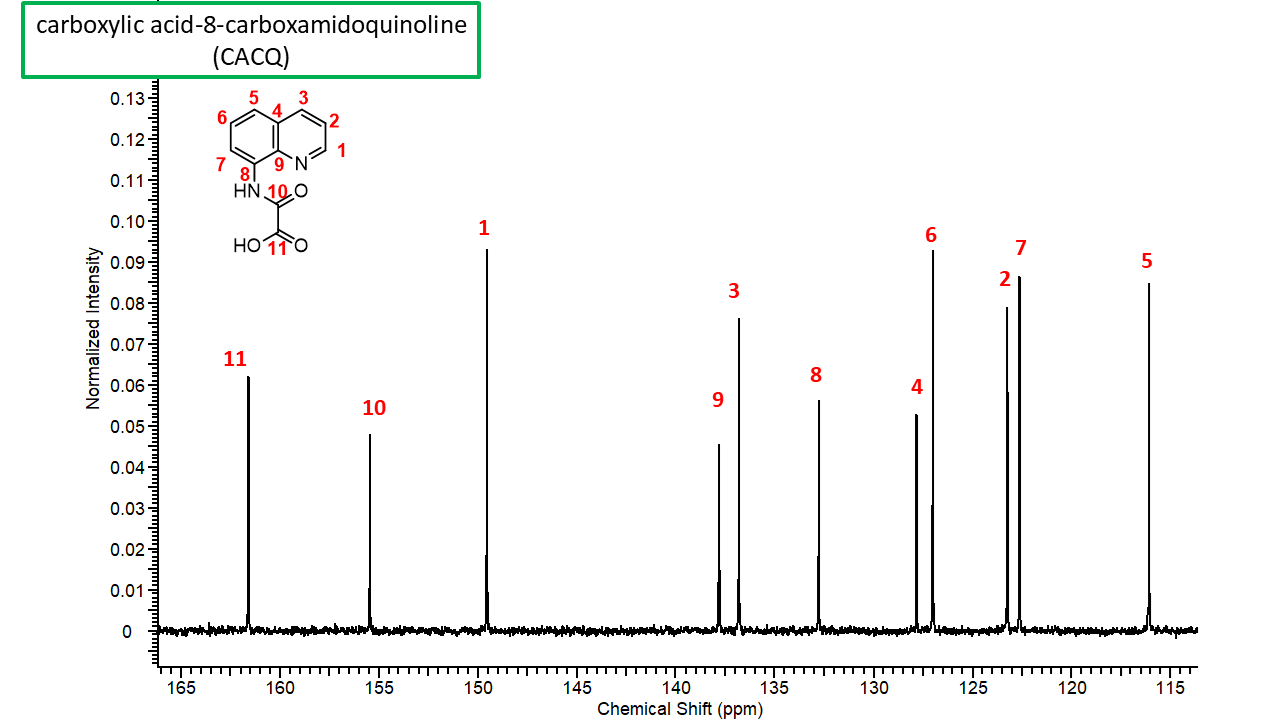


List of the carbon’s signal of CACQ, specifying the chemical shift (δ), and assignation of carbons.

| **Chemical shift (δ)** | **Labelling** |
| --- | --- |
| 162.18 | 11 |
| 157.05 | 10 |
| 149.89 | 1 |
| 138.07 | 9 |
| 132.60 | 3 |
| 128.18 | 8 |
| 127.34 | 4 |
| 124.05 | 6 |
| 122.99 | 2 |
| 117.13 | 7 |
