# Supplementary material for: Electrochemically reduced graphene oxide integrated with carboxylated-8-carboxamidoquinoline: A platform for highly sensitive voltammetric detection of Zn(II) ion by screen-printed carbon electrode
Source: PLoS One. 2025 Feb 7;20(2):e0315974. doi: 10.1371/journal.pone.0315974 (PMC11805387; doi:10.1371/journal.pone.0315974)

**Supporting Information**

**Electrochemically reduced graphene oxide integrated with carboxylated-8-carboxamidoquinoline: A platform for highly sensitive voltammetric detection of Zn(II) ion by screen-printed carbon electrode**

Nur Syamimi Mohamad^1^, Nurul Izzaty Hassan^2^, Choo Ta Goh^1^, Ling Ling Tan^1,^*

**1** Southeast Asia Disaster Prevention Research Initiative (SEADPRI), Institute for Environment and Development (LESTARI), Universiti Kebangsaan Malaysia, 43600 UKM Bangi, Selangor Darul Ehsan, Malaysia

**2** Department of Chemical Sciences, Faculty of Science and Technology, Universiti Kebangsaan Malaysia, 43600 UKM Bangi, Selangor Darul Ehsan, Malaysia

*Author to whom correspondence should be addressed: Ling Ling Tan

E-mail address: lingling@ukm.edu.my

**Table of Contents**

1. ATR-FTIR spectrum of CACQ……………………………………………….......1
2. ATR-FTIR absorption frequencies of CACQ…………………………………......2
3. ESI-MS spectrum of CACQ [C_11_H_8_N_2_O_3_ + H]^+^. Positive mode………………………………………………………..………………….......3

ATR-FTIR spectrum of CACQ


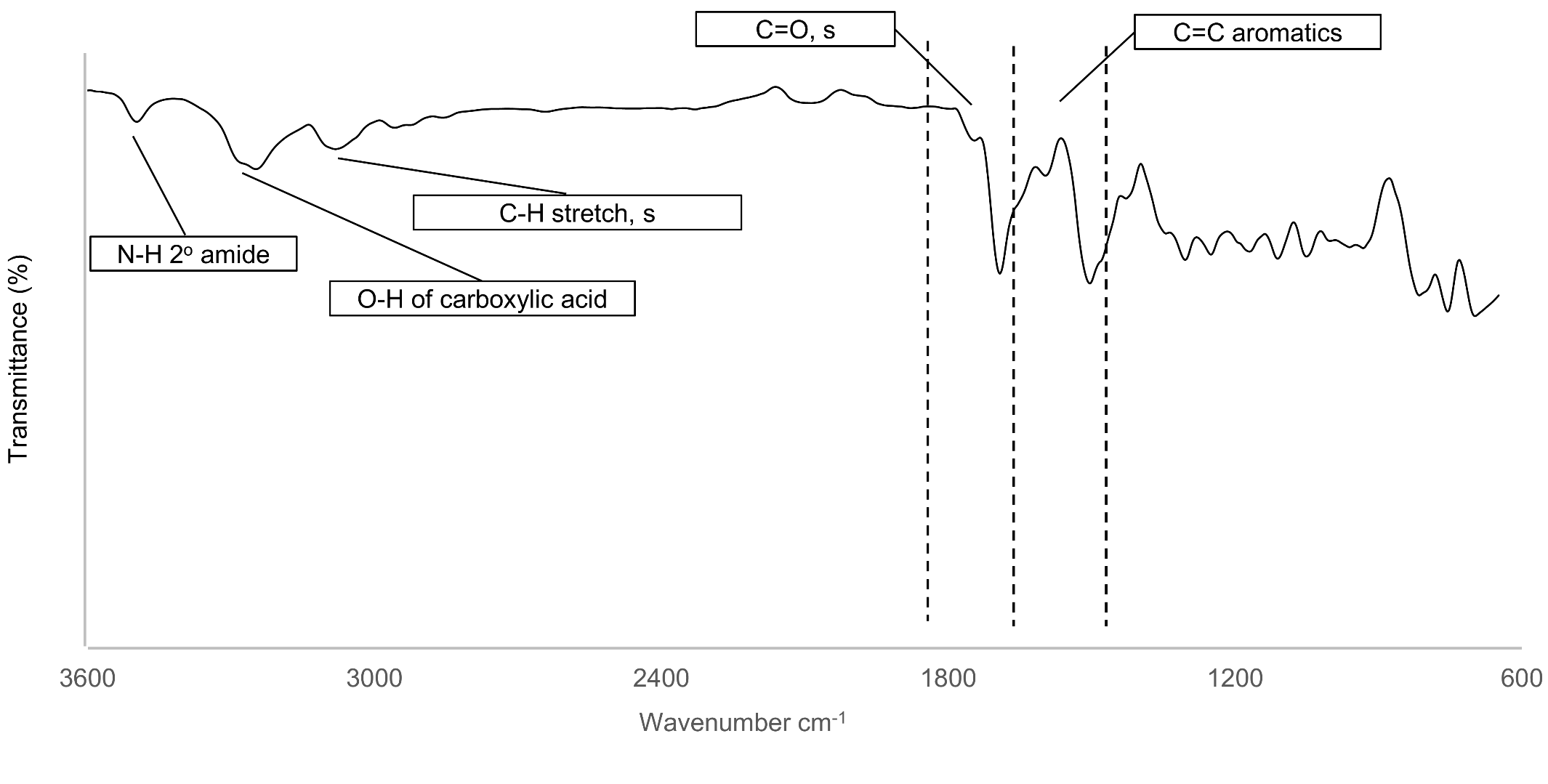


ATR-FTIR absorption frequencies of CACQ

| Functional groups | Wavenumber (cm^-1^) |
| --- | --- |
| C=C aromatic, r | 1502 |
| C=O amide,s | 1688 |
| C=O asid,s | 1688 |
| C-H stretch,as | 3078 |
| O.H., s | 3242 |
| N-H 2º amide, s | 3492 |

* s: strecthing, r: rocking, as: anti-symmetry strecth

ESI-MS spectrum of CACQ [C_11_H_8_N_2_O_3_ + H]^+^. Positive mode.


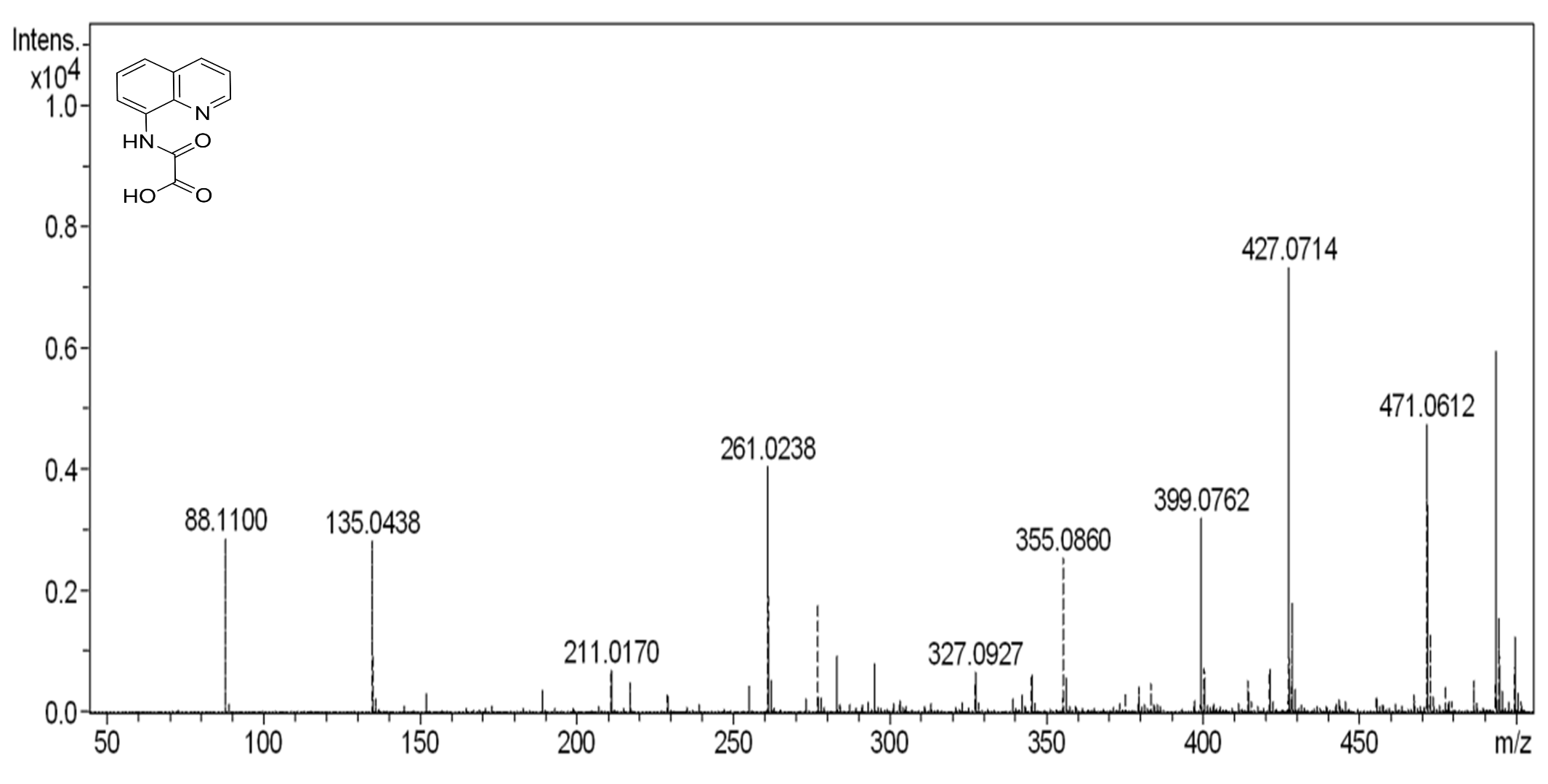

Supplement: S2 Appendix — (I) ATR-FTIR spectrum of CACQ, (II) ATR-FTIR absorption frequencies of CACQ, and (III) ESI-MS spectrum of CACQ [C11H8N2O3 + H]+. Positive mode. (DOCX) [file pone.0315974.s002.docx]
