# Supplementary figures and images for: Electrochemically reduced graphene oxide integrated with carboxylated-8-carboxamidoquinoline: A platform for highly sensitive voltammetric detection of Zn(II) ion by screen-printed carbon electrode
Source: PLoS One. 2025 Feb 7;20(2):e0315974. doi: 10.1371/journal.pone.0315974 (PMC11805387; doi:10.1371/journal.pone.0315974)

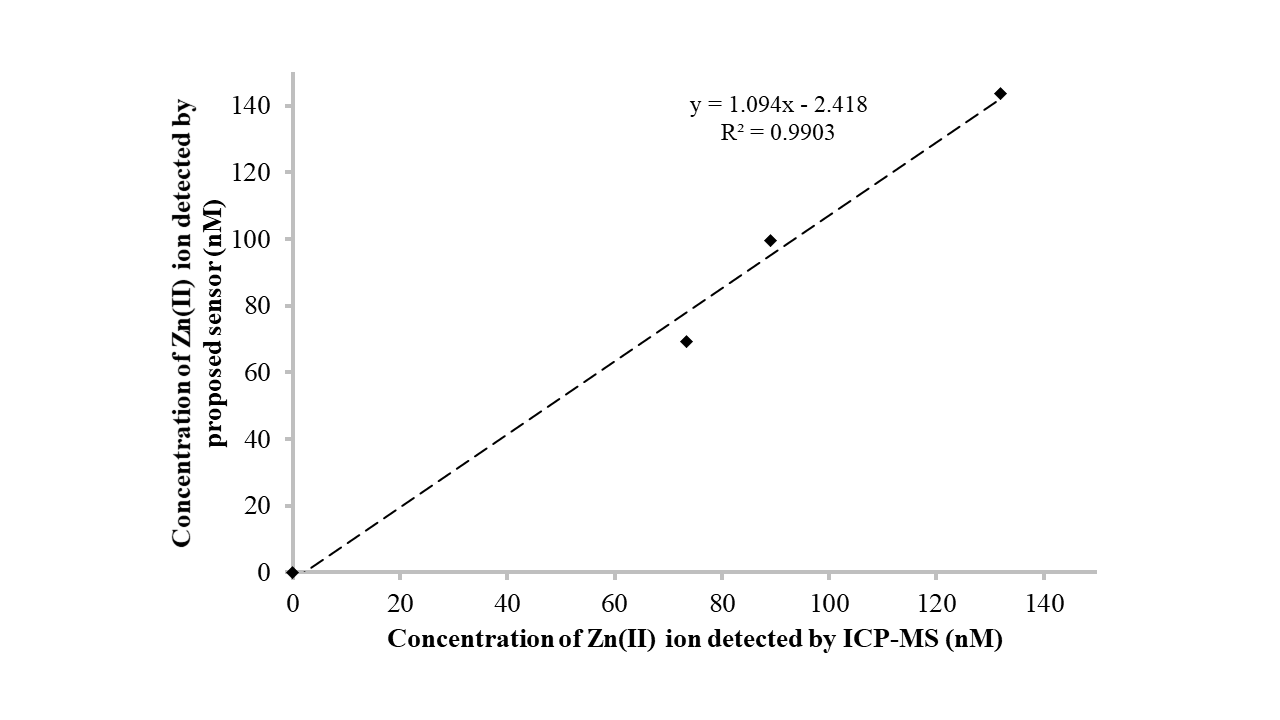

Supplement: S1 Fig — ICP-MS versus proposed sensor. (TIF) [file pone.0315974.s003.tif]

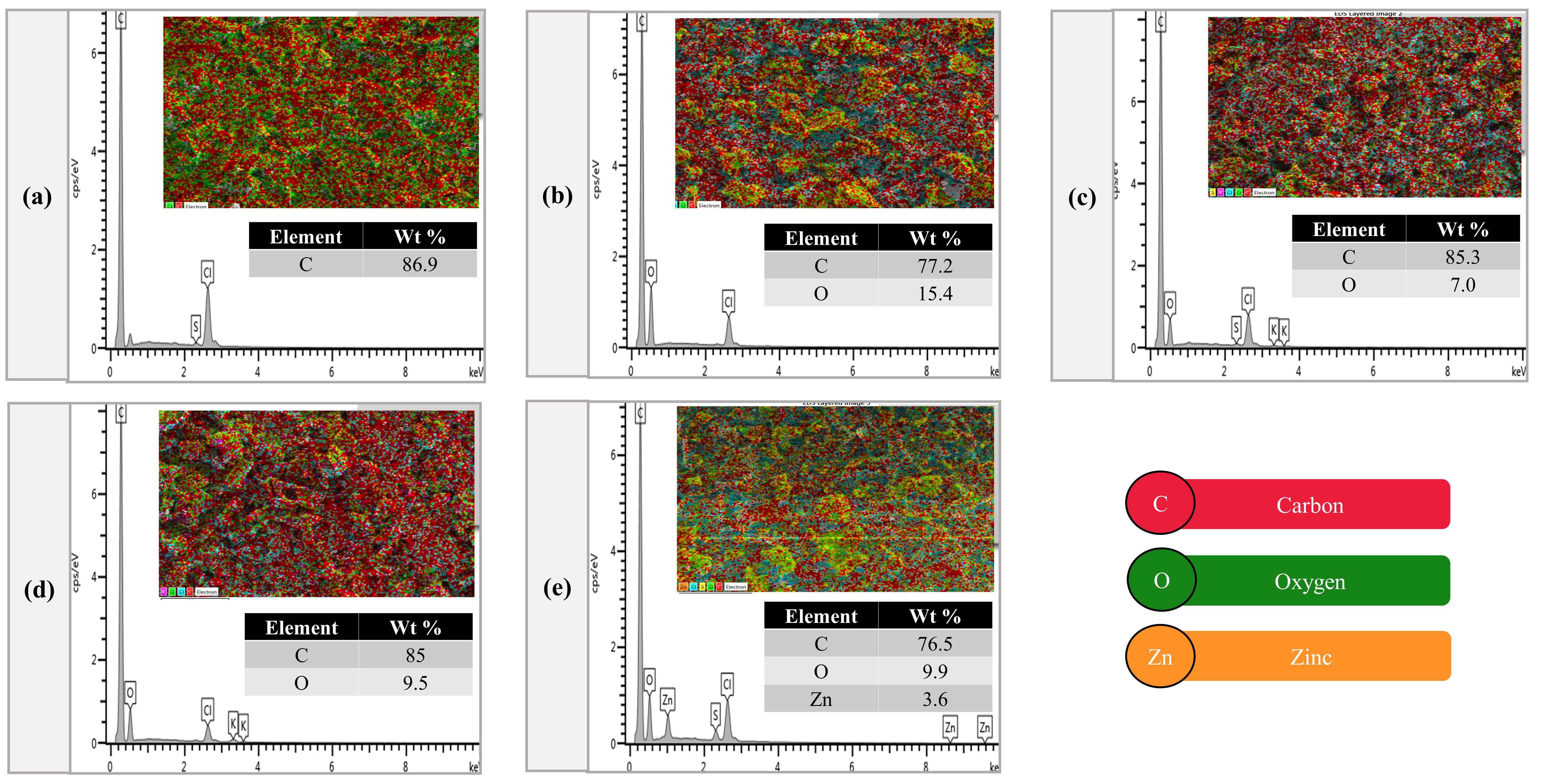

Supplement: S2 Fig — Elemental analysis of (a) unmodified SPCE and modified SPCEs, i.e. (b) GO-SPCE, (c) ErGO-SPCE, (d) CACQ/ErGO-SPCE, and (e) Zn(II)/CACQ/ErGO-SPCE. The EDX mapping’s color representations: red for carbon, green for oxygen, and orange for zinc. (TIF) [file pone.0315974.s004.tif]

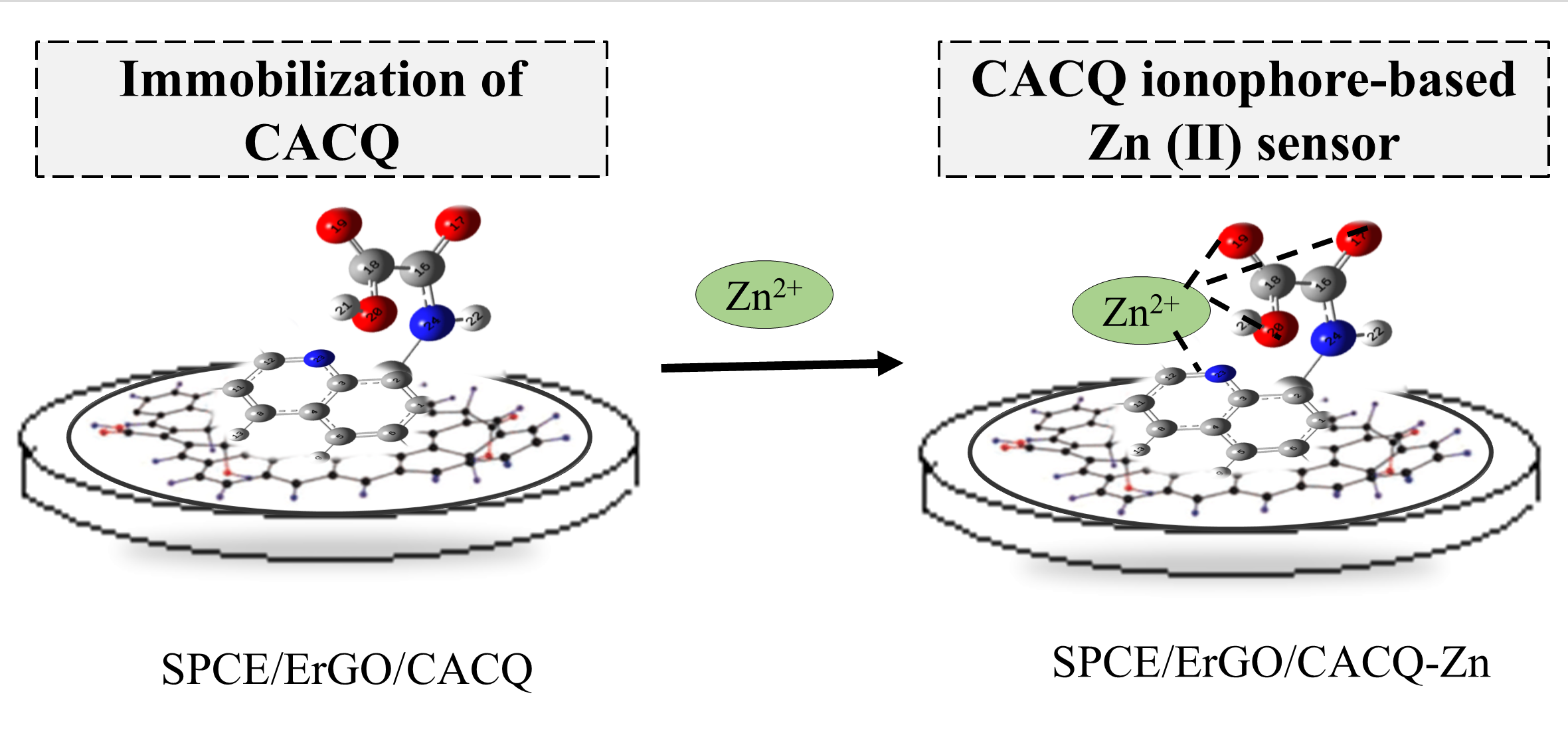

Supplement: S3 Fig — (TIF) [file pone.0315974.s005.tif]
